# Supplementary material for: Association between socioeconomic position and occupational health service utilisation trajectories among young municipal employees in Finland
Source: BMJ Open. 2019 Nov 27;9(11):e028742. doi: 10.1136/bmjopen-2018-028742 (PMC6887011; doi:10.1136/bmjopen-2018-028742)
Supplement: Supplementary data [file bmjopen-2018-028742supp003.pdf]

Web-appendix 3. Multinomial logistic regression on occupational class with reversed order of categories as a determinant of occupational health service (OHS) trajectories among 9762 City of Helsinki employees aged 20-34 years. Results are based on register data covering the years from 2004 to 2017.

	**OHS trajectory comparison**	
	Low/increasing vs. No OHS visits	
	Model 1*	Model 2**
**Men**		
**Occupational class**		
Worker occupations***	1.00	1.00
Semi-professionals	1.02 (0.72-1.44)	1.04 (0.72-1.50)
Managers or professionals	0.65 (0.49-0.88)	0.79 (0.55-1.13)
**Women**		
**Occupational class**		
Worker occupations	1.00	1.00
Semi-professionals	0.83 (0.70-0.99)	0.84 (0.69-1.02)
Managers or professionals	0.67 (0.56-0.79)	0.70 (0.56-0.89)
	Low/decreasing vs. No OHS visits	
	Model 1*	Model 2**
**Men**		
**Occupational class**		
Worker occupations	1.00	1.00
Semi-professionals	1.03 (0.74-1.43)	0.96 (0.68-1.36)
Managers or professionals	0.78 (0.60-1.02)	0.66 (0.47-0.93)
**Women**		
**Occupational class**		
Worker occupations	1.00	1.00
Semi-professionals	1.12 (0.97-1.30)	1.02 (0.86-1.22)
Managers or professionals	0.76 (0.65-0.88)	0.60 (0.48-0.74)
	High/recurrent vs. No OHS visits	
	Model 1*	Model 2**
**Men**		
**Occupational class**		
Worker occupations	1.00	1.00
Semi-professionals	0.70 (0.43-1.15)	0.80 (0.47-1.34)
Managers or professionals	0.21 (0.13-0.38)	0.31 (0.16-0.58)
**Women**		
**Occupational class**		
Worker occupations	1.00	1.00
Semi-professionals	0.81 (0.66-0.99)	0.98 (0.77-1.24)
Managers or professionals	0.38 (0.30-0.49)	0.42 (0.31-0.59)

* Model adjusted for age and language ** Model 1 adjusted for education *** Combined class for routine non-manual employees and manual workers
