# Supplementary material for: Association between socioeconomic position and occupational health service utilisation trajectories among young municipal employees in Finland
Source: BMJ Open. 2019 Nov 27;9(11):e028742. doi: 10.1136/bmjopen-2018-028742 (PMC6887011; doi:10.1136/bmjopen-2018-028742)
Supplement: Supplementary data [file bmjopen-2018-028742supp001.pdf]

Web-appendix 1. Comparison between those included* and those not included* in the present study, men

Men	Total		Included		Excluded	
	N	%	N	%	N	%
**Total**	6413	100.0	2454	100.0	3959	100.0
**Age**						
20 - 24	2170	33.8	618	25.2	1552	39.2
25 - 29	2507	39.1	1077	43.9	1430	36.1
30 - 34	1736	27.1	759	30.9	977	24.7
**Language**						
Finnish	5580	87.0	2154	87.8	3426	86.5
Swedish	274	4.3	81	3.3	193	4.9
Other	501	7.8	193	7.9	308	7.8
**Education**						
Basic education / Lower secondary	4604	71.8	1595	65.0	3009	76.0
Upper secondary	927	14.5	476	19.4	451	11.4
Higher education	882	13.8	383	15.6	499	12.6
**Occupational class**						
Managers or professionals	1331	20.8	594	24.2	737	18.6
Semi-professionals	587	9.2	286	11.7	301	7.6
Routine non-manual workers	1918	29.9	677	27.6	1241	31.3
Manual workers	2043	31.9	897	36.6	1146	28.9
**Working hours per week**						
32–45 h/wk	5003	78.0	1967	80.2	3036	76.7
<32 h/wk	1410	22.0	487	19.8	923	23.3
**Type of employment contract**						
Permanent contract	4603	71.8	2117	86.3	2486	62.8
Other contract type	1810	28.2	337	13.7	1473	37.2

*Included = employed by the City of Helsinki for at least 4 years and complete data on occupational position, Not included= employed by the City of Helsinki less than 4 years or incomplete data on occupational position.
