# Supplementary material for: Association between socioeconomic position and occupational health service utilisation trajectories among young municipal employees in Finland
Source: BMJ Open. 2019 Nov 27;9(11):e028742. doi: 10.1136/bmjopen-2018-028742 (PMC6887011; doi:10.1136/bmjopen-2018-028742)
Supplement: Supplementary data [file bmjopen-2018-028742supp002.pdf]

Web-appendix 2. Comparison between those included* and those not included* in the present study, women

Women	Total		Included		Excluded	
	N	%	N	%	N	%
**Total**	16163	100.0	7308	100.0	8855	100.0
**Age**						
20 - 24	5609	34.7	2252	30.8	3357	37.9
25 - 29	6735	41.7	3152	43.1	3583	40.5
30 - 34	3819	23.6	1904	26.1	1915	21.6
**Language**						
Finnish	14028	86.8	6451	88.3	7577	85.6
Swedish	803	5.0	324	4.4	479	5.4
Other	1137	7.0	448	6.1	689	7.8
**Education**						
Basic education / Lower secondary	8785	54.4	3641	49.8	5144	58.1
Upper secondary	4420	27.3	2319	31.7	2101	23.7
Higher education	2958	18.3	1348	18.4	1610	18.2
**Occupational class**						
Managers or professionals	3674	22.7	1679	23.0	1995	22.5
Semi-professionals	2833	17.5	1538	21.0	1295	14.6
Routine non-manual workers	7561	46.8	3385	46.3	4176	47.2
Manual workers	1875	11.6	706	9.7	1169	13.2
**Working hours per week**						
32–45 h/wk	12678	78.4	5891	80.6	6787	76.6
<32 h/wk	3485	21.6	1417	19.4	2068	23.4
**Type of employment contract**						
Permanent contract	13059	80.8	6422	87.9	6637	75.0
Other contract type	3104	19.2	886	12.1	2218	25.0

*Included = employed by the City of Helsinki for at least 4 years and complete data on occupational position, Not included= employed by the City of Helsinki less than 4 years or incomplete data on occupational position.
