# Supplementary material for: Association between socioeconomic position and occupational health service utilisation trajectories among young municipal employees in Finland
Source: BMJ Open. 2019 Nov 27;9(11):e028742. doi: 10.1136/bmjopen-2018-028742 (PMC6887011; doi:10.1136/bmjopen-2018-028742)
Supplement: Author's manuscript [file bmjopen-2018-028742.draft_revisions.pdf]

BMJ Open

BMJ Open is committed to open peer review. As part of this commitment we make the peer review history of every article we publish publicly available.

When an article is published we post the peer reviewers' comments and the authors' responses online. We also post the versions of the paper that were used during peer review. These are the versions that the peer review comments apply to.

The versions of the paper that follow are the versions that were submitted during the peer review process. They are not the versions of record or the final published versions. They should not be cited or distributed as the published version of this manuscript.

BMJ Open is an open access journal and the full, final, typeset and author-corrected version of record of the manuscript is available on our site with no access controls, subscription charges or pay-per-view fees (http://bmjopen.bmj.com).

If you have any questions on BMJ Open's open peer review process please email info.bmjopen@bmj.com

BMJ Open

Young Employees' Trajectories and Occupational Class Differences in Utilization of Primary Care Services Provided by Occupational Health Service

Journal:	*BMJ Open*
Manuscript ID	bmjopen-2018-028742
Article Type:	Research
Date Submitted by the Author:	09-Jan-2019

Complete List of Authors:	Sumanen, Hilla; South-Eastern Finland University of Applied Sciences, Health care and emergency care; University of Helsinki, Public Health<br>Harkko, Jaako; University of Helsinki, Social Sciences<br>Piha, Kustaa ; University of Helsinki, Public Health<br>Pietilainen, Olli; University of Helsinki, Department of Public Health<br>Rahkonen, Ossi; University of Helsinki, Department of Public Health<br>Kouvonen, Anne; Helsingin Yliopisto, Faculty of Social Sciences; Queen's University Belfast,

Keywords:	health care visits, socioeconomic differences, young adults

SCHOLARONE™
Manuscripts

Young Employees' Trajectories and Occupational Class Differences in Utilization of Primary Care Services Provided by Occupational Health Service

Hilla Sumanen, PhD 1,2

Jaakko Harkko, PhD 3

Kustaa Piha, PhD 2

Olli Pietiläinen, MSc 2

Ossi Rahkonen, Professor 2

Anne Kouvonen, Professor 3, 4, 5

1. South-Eastern Finland University of Applied Sciences, Unit of Healthcare and Emergency care and Unit of Research, Development and Innovation
2. University of Helsinki, Faculty of Medicine, Department of Public Health
3. University of Helsinki, Faculty of Social Sciences
4. SWPS University of Social Sciences and Humanities in Wroclaw
5. Queen's University Belfast, Administrative Data Research Centre – Northern Ireland, Centre for Public Health

Correspondence to: Hilla Sumanen, Department of Public Health, University of Helsinki, Finland, P.O. Box 20 (Tukholmankatu 8B), FIN-00014, University of Helsinki, Finland; hilla.sumanen@helsinki.fi

For peer review only

What is already known about this subject?

This is the first study utilizing longitudinal occupational health service data. All the findings are novel.

What are the new findings?

We identified four occupational health service utilization groups among young employees: “No visits” (50%), “Low/increasing” (18%), “Low/decreasing” (22%) and ”High/recurrent” (10%) use. Lower occupational classes had a higher propensity for “High/recurrent” OHS utilization for both genders.

How might this impact on policy or clinical practice in the foreseeable future?

Preventive measures should be targeted particularly to the trajectory groups of “Low/increasing” and “High/recurrent” in order to intervene early. Occupational health service utilization should be more closely monitored among the two lowest occupational classes.

Running head: Young Employees’ Utilization of Occupational Health Service

For peer review only

Abstract

OBJECTIVES: To identify groups of municipal employees between the ages of 20 and 34 years with distinct utilization trajectories of primary care services provided by occupational health service (OHS), measured as the annual number of OHS visits, and to identify demographic and socio-economic risk factors that distinguish employees in the high utilization trajectory group(s).

METHODS: The present study is a retrospective register-based cohort study. All municipal employees of the City of Helsinki, Finland, aged 20-34 in the Helsinki Health Study (HHS), recruited from 2004 to 2013, with follow-up data for four years were included in the study (n = 10,064). The outcome measure was group-based trajectories of OHS utilization, identified with a group-based trajectory analysis (GBTA). The demographic and socio-economic variables used to predict the outcome were age, first language, educational level and occupational class. The analyses were stratified by gender.

RESULTS: A large proportion of the young employees do not utilize OHS. Trajectory groups of “No visits” (50%), “Low/increasing” (18%), “Low/decreasing” (22%) and ”High/recurrent” (10%) use were identified. We found occupational class differences in OHS utilization patterns showing that lower occupational classes had a higher propensity for “High/recurrent” OHS utilization for both genders.

CONCLUSIONS: Preventive measures should be targeted particularly to the trajectory groups of “Low/increasing” and “High/recurrent” in order to intervene early. In addition, OHS utilization should be closely monitored among the two lowest occupational classes. More research with longitudinal OHS data is needed.

Key terms: Health care visits, Socioeconomic differences, Young adults, Women, Men

Strengths and limitations of this study

- This is the first study utilizing longitudinal occupational health service data
- The sample consisted all of 20-34-year-old employees of the City of Helsinki. The occupational health care policies are same for all these employees and have remained same during the study period of 2004-2017.
- Our study avoids a common limitation of occupational health service studies that they are based on limited samples with data on only those attending the service or those responding the survey questionnaire.
- Limitations of the data include the lack of diagnostic information and lifestyle factors.
- The lack of other primary care visits outside the occupational health service limits the interpretation of the results.

For peer review only

Introduction

Finland has a unique occupational health service (OHS) system with statutory prevention of occupational health hazards (preventive services) and additionally purchased primary care services. OHS may be provided by employer's own OHS units, private clinics or public health centers with specifically educated occupational health physicians, nurses, physiotherapists and psychologists. In Finland, most employers purchase the additional primary care element for their employees. From 2006 to 2016, 91 to 95% of the employees covered by statutory preventive OHS also had an access to primary care paid by their employer and partly subsidized by National Pension Fund [1]. OHSs are free for employees at the point of delivery.

The utilization of OHS in Finland or elsewhere has been only scarcely studied, and previous studies using longitudinal data do not exist. There are few previous Finnish studies concentrating on the OHS primary care visits from the viewpoint of service use over the course of 6 to 12 months. A recent study [2] with data from a large private Finnish OHS provider investigated the top 10% frequent attenders in primary care services in 2015. The results showed that frequent attendance was associated with female gender, being employed by a medium or a large company, working in the manufacturing industry, public administration or in health and social care services. In an earlier study with survey data (N=1636) from the Finnish working-age population [3], 57% of Finnish employees covered by the OHS primary care visited either occupational health physician or nurse due to any illness during the 6-month period. In that study, those visits were strongly associated with chronic illness impacting occupational health and work ability. Both previous studies utilizing Finnish OHS data acknowledge the lack of research focusing to these unique services provided for the working population and identify the need for further study to identify service development needs and possibilities.

The current study utilizes longitudinal data from the own OHS unit of Finland's largest employer. The City of Helsinki offers same OHSs with primary care for all its employees (n=~38 000 per

For peer review only

year) with no cost for the employees. The City of Helsinki employees have been the focus of the Helsinki Health Study [4] since 2000, but this is the first study using their OHS data. Our focus is on the younger employees and their OHS utilization, as previous studies have shown that they have a high prevalence of sickness absence (SA) [5] and there are already large socioeconomic differences in SA apparent in the younger age groups [6-7]. Reducing SA is high on employers' agenda. Service utilization information is important for planning preventive actions via targeted interventions or improving case management protocols [8-9] arranged by the OHS. According to extensive Finnish and international evidence socioeconomic differences in SA are large among employees [10-21], thus the differences in OHS utilization is feasible to monitor from the socioeconomic viewpoint and to be able to identify groups for interventions.

In our study, we aimed to identify developmental trajectories in OHS primary care service utilization in the municipal employees of the City of Helsinki between the ages of 20 and 34 years from 2004 to 2017. In the second stage of the analysis we aimed to identify demographic and socio-economic determinants of belonging to different trajectory groups. We tested the hypothesis that lower occupational classes have a higher risk of high OHS utilization, when differences in terms of personal characteristics (age, education, first language) are brought into the analysis.

Methods

This is a retrospective register-based cohort study. The study is a part of the Helsinki Health Study on health and well-being among employees of the City of Helsinki, Finland [4]. The study covered all Helsinki City employees aged 20-34 at the beginning of their first work contract with the City (N = 23,388) between 04/06/2004 and 04/19/2013. For each employee the follow-up started from their initial recruitment and only employees with employment record for four years were retained in the

For peer review only

present study (n = 10,064). The length of the follow-up was measured as calendar days in employment.

OHS utilization was the outcome of the study. It was measured by the annual number of outpatient visits ranging from 0 to 103. The demographic and socioeconomic variables used to predict the outcome were age, first language, educational level and occupational class. The analyses were stratified by gender.

Age was categorized into three groups: 20-24, 25-29 and 30-34-year-olds. First language was categorized as: Finnish, Swedish, Other. Education was classified into three levels: higher education (a Master's or a doctor's degree), upper secondary (a Bachelor's degree), and lower secondary (upper-secondary school, vocational school) or basic education (comprehensive school) . Occupational class was measured with four classes: "managers and professionals" (e.g. teachers and physicians), "semi-professionals" (e.g. nurses and foremen), "routine non-manuals" (e.g. clerical employees and childminders) and "manual workers" (e.g. technical and cleaning staff).

The employer's personnel and occupational health care registers were used to obtain socio-demographic characteristics and information on OHS use. Educational level was obtained from annually updated Statistics Finland's registry of completed education and degrees, and was linked to the City of Helsinki personnel register.

Ethics

The study follows the Helsinki Health Study protocol in line with the University of Helsinki's guidelines and data legislation. The ethics committees of the Department of Public Health, the University of Helsinki and the health authorities of the City of Helsinki have approved the HHS study. The City of Helsinki and register holders have given permission for data linkage.

For peer review only

Patient and Public Involvement statement

No Patient or Public Involvement.

Statistical methods

Group-based trajectory modeling (GBTM) with Stata's *traj* command [22] was applied to identify clusters of individuals, or trajectory groups, with similar developmental trajectory on OHS utilization. The method assigns a subject to a trajectory group by assessing the probability of group membership. The count variables were assumed to be Poisson-distributed and zero inflated Poisson models were applied. The ideal number of trajectory groups and trajectory shapes were assessed by four criteria suggested by the existing literature: Bayesian information criteria (BIC), posterior probabilities of trajectory group membership higher than .70, sizes of trajectory groups larger than 5% and a distinct interpretability of the identified trajectory groups [23,24]. Subsequently, multinomial logistic regression models using Stata's *mlogit* command were applied to investigate the role of occupational class as a predictor of the trajectory group membership. In a two-step-analysis, estimates are given for occupational class, first, adjusted for age and first language and, second, additionally adjusted for education. The trajectory cluster indicating the lowest health care utilization was defined as the reference group in the analysis. The results are given as relative risk ratios (RRR) with their 95% confidence intervals (CIs). All statistical analyses were performed with Stata 15.

Results

Descriptive results

The study sample included 10,064 the City of Helsinki employees aged between 20 and 34 at the beginning of the follow-up. 27% of the employees were men and 73% women. Among men, the

For peer review only

yearly average of OHS visits was 1.03 (SD, 1.44) during the mean of 1341 days of follow-up. Among women, the yearly average was 1.16 (SD, 1.50) OHS visits in the mean of 1328 days. Of the subjects of the study, 2272 (23%) were managers or professionals, 1824 (19%) semi-professionals, 4064 (42%) routine non-manual workers, and 1602 (16%) manual workers (Tables 1-2). Whereas managers/professionals had an equal gender distribution, semi-professionals and routine non-manual workers were more often women whereas men constituted the majority of manual workers. Occupational class was closely linked to educational attainment in both genders.

In trajectory analysis, a trajectory model consisting of four distinct trajectories including 1 trajectory with a linear, 1 with a quadratic, and 2 with a cubic shape showed the best fit using the BIC criterion (Figure 1). The largest identified trajectory group "No visits" (n = 5,106, 50%) represents those with less than 0.2 annual OHS visits over the four years of follow-up. There were two intermediate groups. The group labeled "Low/increasing" (n = 1,744, 18%) is characterized by low number of visits during the first two years followed by a slight increase in visits during the next two years. The group "Low/decreasing" (n = 2,238, 22%) follows a similar pattern as the group 2, but in a reverse order. The members of the both low groups averaged 1.5 annual OHS visits during the follow-up. The "High/recurrent" group (n = 976, 10%) consists of employees characterized by high levels of OHS visits, with an average of 4.6 visits per year, from the initiation of the employment to the end of the follow-up. The mean assignment probabilities were 0.93 for the "No visits", 0.81 for the "Low/increasing", 0.82 for the "Low/decreasing", and 0.92 for the "High/recurrent" trajectory groups, indicating a good model fit to the data. Of men, 54% belonged to the "No visits" trajectory and 8.4% to the "High/recurrent" trajectory group, whereas the corresponding figures for women were 50% and 10%, indicating a higher propensity for women to belong to the "High/recurrent" trajectory group. The assignment of the members of different occupational classes to different OHS trajectories followed the socio-economic gradient. Of managers or professionals, 59% belonged to the "No visits" trajectory and 5% to the

For peer review only

"High/recurrent" trajectory. The corresponding figures for semi-professionals were 48% and 10%, for routine non-manual workers 48% and 11%, and for manual workers 49% and 13%, respectively.

*** Figure 1 ***

*** Table 1 ***

*** Table 2 ***

OHS utilization trajectories by occupational class

Occupational class was a strong independent predictor for the OHS utilization trajectories, as demonstrated in Table 3. The likelihood of belonging to the "High/recurrent" trajectory was increased for those being in a lower occupational class. For both women and men, the risk for belonging to the "High/recurrent" trajectory was highest for manual workers, followed by routine non-manual workers and semi-professionals.

The independent effect of occupational class remained after adjustment for all covariates including age, first language and education. The association was most evident in the "High/recurrent" trajectory. The relative risk for this group membership was 2.92 (95% CI, 1.48 – 5.74) for male routine non-manual workers and 3.56 (95% CI, 1.83 – 6.92) for male manual workers. The corresponding figures for women were 2.28 (95% CI, 1.65 – 3.15) and 2.71 (95% CI, 1.85 – 3.97), respectively. The results indicate that a proportion of the association between occupational class and belonging to the "High/recurrent" trajectory is dependent on the lower educational attainment of the members of the lower occupational classes. The results comparing the two low trajectories with the "No visits" OHS trajectory were less clear in terms of statistical significance. Whereas all estimates but one in "Low/decreasing" vs "No visits" comparison remained statistically significant after full

For peer review only

adjustment, four out of “Low/increasing” vs “No visits” comparisons become statistically non-significant in the final model. The observed excess risks generated by occupational class were thus smaller in both “Low” trajectories compared to “High/recurrent” trajectory. Notably, the relative risks related to the membership of these middle trajectories were not manifested in a dose-exposure manner as was the case with the “High/recurrent” trajectory.

*** Table 3 ***

Sensitivity analyses

To assess the extent the parameter estimates were sensitive to potential errors in model specification and data, two types of sensitivity analyses were performed. First, we reproduced estimates from the original data with bootstrap resampling (1000 replications). Second, we reproduced the results with logistic regression analyses defining the high-utilization group as those whose total number of OHS visits during the follow-up period was 10 or more (n=1,484) was compared with those with no or just one visit (n= 3,993). These sensitivity analyses indicated robustness of our inference about the relationship between occupational class and OHS utilization trajectories.

Discussion

In this study we identified developmental trajectories and socioeconomic differences in OHS primary care service utilization among 20 to 34-year-old employees of the City of Helsinki from 2004 to 2017. Our key results were: 1) Half of the young employees did not use occupational health care services to any considerable extent; 2) Higher occupational classes utilized less OHS; 3) Four trajectory groups, that is, “No visits”, ”Low/increasing”, “Low/decreasing” and “High/recurrent”,

For peer review only

were identified. 4) The trajectory group of "High/recurrent" included a larger number of lower class workers especially among men, and the differences were large also among women. 5) Occupational class differences in "Low/decreasing" group were evident in both genders. 6) Only in women there were some occupational class differences in belonging to the trajectory group of "Low-increasing OHS utilization".

Our results highlight the significance of socioeconomic gradient in the OHS utilization that was visible both in men and women. The percentage of those who had no visits was the highest among managers and professionals and the proportion of no visits decreased with when going down the occupational class ladder. Respectively, high and recurrent use was smallest among managers and professionals, and increased with decreasing occupational class, this type of use being the most common among manual workers. A larger proportion of men had no visits at all in each occupational class, thus the women used the health services more, in line with earlier findings [2]. In a similar way, the high and recurrent use was higher among women than among men. In the present context, primary care visits can be interpreted as an indicator of incidence of acute illnesses, as the Finnish OHS system distinguish visits related to occupational health hazards. The present results concerning primary care visits are in line with previous findings from our own and other studies showing the socioeconomic differences in sickness absence among employees and the gender differences in sickness absence, i.e. women having more absences than men [7,10,14,18,21]. It can be assumed that large number of OHS visits precede sickness absence as also indicated by Bergh et al. [25].

Stratified analyses indicated gender differences in OHS utilization. According to our results, among men the occupational class differences disappeared after full adjustment in the trajectory group of "Low/increasing". This implies that the initial differences are associated with the type of work tasks. In contrast, after full adjustment, among women the employees in the two lowest occupational classes had a higher risk for belonging to this trajectory group. In the trajectory group

For peer review only

of “Low/decreasing” the differences were initially similar among the three lowest occupational classes. However, the differences modestly increased after full adjustment, implying that there are several factors associated with the low OHS utilization. This was seen among both genders.

The trajectory group of “High/recurrent” is perhaps the most interesting group alongside with the “Low/increasing” group in terms of costs and possible preventive opportunities. According to our results the occupational class differences in this group are steep especially among men, and also large among women. After full adjustment the differences decreased more in men, suggesting that the initial differences are more associated with work tasks among them. However, the differences remained high in both genders after adjustments. Studies regarding socioeconomic differences in sickness absence have also found that the differences are steeper among men [7,17,18], but the former studies mostly concentrate on older employees.

The differences in physical and psychosocial demands between occupational classes are important to take into account when interpreting the results. Manual workers have more physically demanding jobs, which may affect their need for primary care services. Adverse working conditions may cause ill-health and need of health services as milder even health difficulties may prevent these employees from working. Employees in higher occupational classes typically have more complex and mentally demanding jobs [26]. In studies examining the socioeconomic differences in sickness absence, physical working conditions have been found to be the strongest explanatory factor [12,16]. However, employees in our study are fairly young and thus adverse physical or psychosocial working conditions might not have yet affected their health, as health-related effects usually increase with age. In addition, the unique OHS system where the visits associated with occupational health hazards (preventive services) are recorded separately from primary care visits may contribute to the differences seen in our results. For example visits with more chronic work-related reason are usually not recorded as primary care visits. Thus, the overall utilization of OHS requires further research.

For peer review only

Our study indicates that service use patterns might recognize vulnerable groups more precisely than just belonging to certain occupation or occupational class may do. Despite this, the two lowest occupational classes may need extra attention based on their OHS utilization patterns. Case management protocols are essential in coordinating patient-centered care path which also saves costs [8,9,27]. Among younger employees, timely treatment is highly important, as it might prevent the worsening of their condition. OHS should identify those employees who use services a lot.

Previous studies using OHS data are lacking, but some comparisons can be made with the studies investigating frequent attenders in primary care. Frequent attenders in primary care in the general population have been studied particularly in Netherlands and in Sweden using questionnaire surveys and record linkage. These studies had participants from a wide range of sociodemographic backgrounds and they consider only visits to general practitioners, thus their direct comparability to OHS utilization is difficult. In addition, the definition of frequent attender varies between studies [28]. However, previous studies have found out that frequent attenders have multiple reasons for presenting [29], but overall chronic illnesses [28], somatic diseases and symptoms [30,31] and especially psychiatric problems [30] have been associated with more frequent primary care service use. Frequent attenders have more health discomfort, low mastery, and they may be more vulnerable for stressful life events due to inadequate coping strategies [25,32,33]. Frequent attenders are a high-risk group for long-term sickness absence and disability pension [25]. According to two previous Dutch studies one out of every seven 1-year frequent attenders becomes persistent frequent attender and six out of seven are transient frequent attenders [30,34]. Based on this previous evidence the inclusion of diagnostic information would be important in future studies of OHS utilization. However, from methodological viewpoint both the Dutch and Swedish research groups point out that age should be taken into account when studying the frequent attenders, as not only the reasons for high service utilization but also what constitutes high use highly differ by age group [31,35].

For peer review only

Methodological considerations

The registers used in this study are reliable and comprehensive. We focused on all occupational groups within the municipality and the sample consisted all of 20-34-year-old employees within this organization. The occupational health care policies are same for all these employees and have remained same during the study period of 2004-2017. Our study avoids a common limitation of OHS studies that they are based on limited samples with data on only those attending the service (e.g. 2) or those responding the survey questionnaire [3]. Another advantage of this study is that we could make inferences based on longitudinal cohort data instead of relying on just cross-sectional evidence. Limitations of the data include the lack of diagnostic information, lifestyle factors and other primary care visits outside the OHS.

The OHS system in Finland is unique, thus comparisons to other countries is difficult. Even within Finland, different employers can have different policies in terms of provision of primary care services. Nevertheless, our results can be broadly generalized to the Finnish public sector.

The present study is to our knowledge the first one that used longitudinal latent class analysis aiming to capture OHS utilization as a complex longitudinal phenomenon. GBTM approach mixes the application of formal statistical criteria and subjective evaluation in model fitting [23]. One of its strength is that it allowed us to identify high OHS utilization over time. It is a limitation that those who left the City of Helsinki within the first four years of their employment were lost to follow-up. Another benefit is that GBTM is capable of identifying different OHS trajectories within subjects that appear similar in terms of summary statistics. In this study we were able to distinguish between two "low" trajectories, which may allow for better planning of targeted prevention measures. However, we want to highlight that by this methodology it cannot be ascertained that the observed subgroups are distinct population subgroups. As in case of any latent trajectory class analyses, there is a possibility that the data could be interpreted as homogenous but non-normal

For peer review only

[36]. We find, however, the obtained groups to be realistic and the results applicable in terms of real-life interpretations.

Conclusions

We used GBTM for distinguishing four different developmental trajectories in OHS primary care service utilization among 20-34-year-old employees of the City of Helsinki. Occupational class differences exist in the utilization development trajectories. A large proportion of the young employees do not use OHS primary care services and non-use is most common among the highest occupational class. Especially trajectories where the utilization has grown or been high throughout the follow-up had large occupational class differences, which followed the socioeconomic gradient. Identifying high utilization patterns is important as ten per cent of employees that may be labeled high and recurrent users account for 40% of the all OHS consultations.

According to our results preventive measures should be considered among the trajectory groups of "Low/increasing" and "High/recurrent". In addition, attention should be paid to the two lowest occupational classes, and their OHS utilization should be closely monitored by the occupational health care in order to identify those in need for extra support. OHS utilization requires more longitudinal research. Case management protocols should be further developed.

Author contributions

HS, AK & JH designed the study. HS mainly wrote the manuscript with contributions and comments from all the other authors, JH did the statistical analyses and wrote the results section and OP commented those. All the authors have approved the final version of the manuscript.

For peer review only

Conflicts of Interest & Funding

Authors declare no conflicts of interest. This study was funded by the Finnish Work Environment Fund (grant 117321) and the Academy of Finland (grant 315343). AK was funded by the Economic and Social Research Council (ESRC) (grant ES/L007509/1). OR was funded by the Academy of Finland (grant 1294514). The funders had no involvement to the preparation of the manuscript.

Data sharing statement

No additional data available.

For peer review only

References

1. Kelasto-reports. 2018. Selected data for report Occupational health care for employers: Number of persons covered and reimbursements paid out. http://raportit.kela.fi/ibi_apps/WFServlet (accessed 8 Jun 2018).

2. Reho T, Atkins S, Talola N, Viljamaa M, Sumanen M, Uitti J. Frequent attenders in occupational health primary care: A cross-sectional study. Scand J Public Health 2018;1:1403494818777436.

3. Kimanen A, Rautio M, Manninen P, Räsänen K, Husman P, Husman K. Primary care visits to occupational health physicians and nurses in Finland. Scand J Public Health 2011;39:525-32.

4. Lahelma E, Aittomäki A, Laaksonen M, Lallukka T, Martikainen P, Piha K, et al. Cohort Profile: The Helsinki Health Study. Int J Epidemiol 2013;42:722–30.

5. Sumanen H, Pietiläinen O, Lahti J, Lahelma E, Rahkonen O. Sickness absence among young employees: trends from 2002 to 2013. J Occup Health 2015;57:474-81.

6. Sumanen H, Lahelma E, Lahti J, Pietiläinen O, Rahkonen O. Educational differences in sickness absence trends among young employees from 2002 to 2013 in Helsinki, Finland. BMJ Open 2016; 6:e008550.

7. Sumanen H, Lahelma E, Pietiläinen O, Rahkonen O. The Magnitude of Occupational Class Differences in Sickness Absence: 15-Year Trends among Young and Middle-Aged Municipal Employees. Int J Environ Res Public Health 2017;14:E625.

8. AAOHN. Case Management: The Occupational and Environmental Health Nurse Role. Workplace Health Saf 2013;61:8.

9. Robinson M, Fisher TF, Broussard K. Role of Occupational Therapy in Case Management and Care Coordination for Clients With Complex Conditions. Am J Occup Ther 2016;70:7002090010p1-7002090010p6.

10. Piha K, Laaksonen M, Martikainen P, Rahkonen O, Lahelma E. Interrelationships between education, occupational class, income and sickness absence. Eur J Public Health 2010;20:276-80.

11. Piha K, Martikainen P, Rahkonen O, Roos E, Lahelma E. Trends in socioeconomic differences in sickness absence among Finnish municipal employees 1990-99. Scand J Public Health 2007;35:348-55.

12. Laaksonen M, Piha K, Rahkonen O, Martikainen P, Lahelma E. Explaining occupational class differences in sickness absence: Results from middle-aged municipal employees. J Epidemiol Community Health 2010;64:802–7.

13. Sumanen H, Pietiläinen O, Lahti J, Lahelma E, Rahkonen O. Interrelationships between education, occupational class and income as determinants of sickness absence among young employees in 2002-2007 and 2008-2013. BMC Public Health 2015;15:332.

For peer review only

14. Pekkala J, Blomgren J, Pietiläinen O, Lahelma E, Rahkonen O. Occupational class differences in diagnostic-spesific sickness absence: A register-based study in the Finnish population. BMC Public Health 2017;17:670.

15. Feeney A, North F, Head J, Canner R, Marmot M. Socioeconomic and sex differentials in reason for sickness absence from the Whitehall II study. Occup Environ Med 1998;55:91-8.

16. Christensen K, Labriola M, Lund T, Kivimäki M. Explaining the social gradient in long-term sickness absence: A prospective study of Danish employees. J Epidem Community Health 2008;62:181–3.

17. Hansen H, Ingebrigtsen T. Social class and sickness absence in Norway. Acta Sociologica 2008;51:309–27.

18. Löve J, Hensing G, Holmgren K, Toren K. Explaining the social gradient in sickness absence: A study of a general working population in Sweden. BMC Public Health 2013;13:545-2458-13-545.

19. Vahtera J, Virtanen P, Kivimäki M, Pentti J. Workplace as an origin of health inequalities. J Epidemiol Community Health 1999;53:399-407.

20. Morikawa Y, Martikainen P, Head J, Marmot M, Ishizaki M, Nakagawa H. A comparison of socio-economic differences in long-term sickness absence in a Japanese cohort and a British cohort of employed men. Eur J Public Health 2004;14:413-6.

21. Kaikkonen R, Härkänen T, Rahkonen O, Gould R, Koskinen S. Explaining educational differences in sickness absence: a population-based follow-up study. Scand J Work Environ Health 2015;41:338-46.

22. Jones BL, Nagin DS. 2012. A Stata Plugin for Estimating Group-Based Trajectory Models. https://ssrc.indiana.edu/doc/wimdocs/2013-03-29_nagin_trajectory_stata-plugin-info.pdf (accessed 1 Jun 2018)

For peer review only

23. Nagin D. 2005. Group-Based Modeling of Development. Cambridge, Mass: Harvard University Press.

24. Nagin DS, Odgers CL. Group-Based Trajectory Modeling in Clinical Research. Annu Rev Clin Psychol 2010;6:109–38.

25. Bergh H, Baigi A, Månsson J, Mattsson B, Marklund B. Predictive factors for long-term sick leave and disability pension among frequent and normal attenders in primary health care over 5 years. Public Health 2007;121:25-33.

26. Nieuwenhuijsen K, Verbeek JH, de Boer AG, Blonk RW, van Dijk FJ: Predicting the duration of sickness absence for patients with common mental disorders in occupational health care. Scand J Work Environ Health 2006;32:67-74.

27. Golden R. The importance of collaboration. CMSA Today 2015;4:21-2.

28. Vedsted P, Christensen MB. Frequent attenders in general practice care: a literature review with special reference to methodological considerations. Public Health 2005;119:118-37.

29. Smits FT, Brouwer HJ, Schene AH, van Weert HC, Ter Riet G. Is frequent attendance of longer duration related to less transient episodes of care? A retrospective analysis of transient and chronic episodes of care. BMJ Open 2016;6:e012563.

30. Smits FT, Brouwer HJ, ter Riet G, van Weert HC. Epidemiology of frequent attenders: a 3-year historic cohort study comparing attendance, morbidity and prescriptions of one-year and persistent frequent attenders. BMC Public Health 2009:9;36.

31. Bergh H, Marklund B. Characteristics of frequent attenders in different age and sex groups in primary health care. Scand J Prim Health Care 2003;21:171-7.

32. Bergh H, Baigi A, Fridlund B, Marklund B. Life events, social support and sense of coherence among frequent attenders in primary health care. Public Health 2006;120:229-36.

For peer review only

33. Smits FT, Brouwer HJ, Zwinderman AH, Mohrs J, Schene AH, van Weert HC, ter Riet G. Why do they keep coming back? Psychosocial etiology of persistence of frequent attendance in primary care: a prospective cohort study. J Psychosom Res 2014;77:492-503.

34. Smits FT, Brouwer HJ, van Weert HC, Schene AH, ter Riet G. Predictability of persistent frequent attendance: a historic 3-year cohort study. Br J Gen Pract 2009;59:e44-50.

35. Smits FT, Mohrs JJ, Beem EE, Bindels PJ, van Weert HC. Defining frequent attendance in general practice. BMC Fam Pract.2008;15:21.

36. Bauer DJ, Curran PJ. Distributional Assumptions of Growth Mixture Models: Implications for Overextraction of Latent Trajectory Classes. Psychological Methods 2003;8:338-63.

For peer review only

Table 1. Descriptive statistics of the four occupational classes among 2,678 male The City of Helsinki employees aged 20-34 years. Results are based on register data covering the years from 2004 to 2017.

	**Total**				**Occupational class**							
			**OHS visits p.a.**		***Managers or professionals***		***Semi-professionals***		***Routine non-manual workers***		***Manual workers***	
	N	% / (sd.)	N	1 / 1 000	*n*	*% / (sd.)*	*n*	*% / (sd.)*	*n*	*% / (sd.)*	*n*	*% / (sd.)*
**Total**	2454	100.0	2528	1030.3	594	100.0	286	100.0	677	100.0	897	100.0
The length of the follow-up in days, average (sd.)	1341	(252)			1371	(204)	1382	(191)	1290	(292)	1348	(260)
OHS visits per annum, 1 / 1000 (sd.)	1030.3	(1435.3)			705.4	(939.4)	996.5	(1151.3)	1121.5	(1482.1)	1187.3	(1695.8)
**Outcome: Trajectory group**												
1. No OHS visits	1337	54.5	225	168.5	374	63.0	148	51.7	349	51.6	466	52.0
2. Low/increasing	420	17.1	626	1489.3	91	15.3	54	18.9	131	19.4	144	16.1
3. Low/decreasing	496	20.2	728	1468.2	113	19.0	63	22.0	138	20.4	182	20.3
4. High/recurrent	201	8.2	949	4722.6	16	2.7	21	7.3	59	8.7	105	11.7
Covariates												
**Age (years)**												
20 - 24	618	25.2	684	1107.2	23	3.9	38	13.3	221	32.6	336	37.5
25 - 29	1077	43.9	996	924.6	299	50.3	152	53.1	296	43.7	330	36.8
30 - 34	759	30.9	848	1117.6	272	45.8	96	33.6	160	23.6	231	25.8
**First language**												
Finnish	2154	87.8	2219	1029.9	499	84.0	272	95.1	589	87.0	794	88.5

For peer review only

Swedish	81	3.3	36	438.3	47	7.9	2	0.7	24	3.5	8	0.9
Other	193	7.9	256	1327.7	24	4.0	12	4.2	62	9.2	95	10.6
**Education**												
Basic education / Lower secondary	1595	65.0	1807	1132.8	136	22.9	115	40.2	530	78.3	814	90.7
Upper secondary	476	19.4	453	951.2	126	21.2	149	52.1	130	19.2	71	7.9
Higher education	383	15.6	269	701.7	332	55.9	22	7.7	17	2.5	12	1.3

Table 2. Descriptive statistics of the four occupational classes among 7,386 female The City of Helsinki employees aged 20-34 years. Results are based on register data covering the years from 2004 to 2017.

	Total				Occupational class							
			OHS visits p.a.		*Managers or professionals*		*Semi-professionals*		*Routine non-manual workers*		*Manual workers*	
	N	% / (sd.)	N	1 / 1 000	*n*	*% / (sd.)*	*n*	*% / (sd.)*	*n*	*% / (sd.)*	*n*	*% / (sd.)*
**Total**	7308	100.0	8470	1158.9	1678	100.0	1538	100.0	3387	100.0	705	100.0
The length of the follow-up in days, average (sd.)	1328	(254)			1326	(244)	1375	(202)	1310	(272)	1322	(281)
OHS visits per annum, 1 / 1000 (sd.)	1158.9	(1499.2)			850.9	(1114.0)	1141.7	(1357.4)	1268.7	(1627.4)	1402.5	(1808.0)
**Outcome: Trajectory group**												
1. No OHS visits	3623	49.6	644	177.7	972	57.9	734	47.7	1599	47.2	318	45.1
2. Low/increasing	1281	17.5	1922	1500.2	264	15.7	249	16.2	627	18.5	141	20.0
3. Low/decreasing	1651	22.6	2455	1487.0	347	20.7	397	25.8	759	22.4	148	21.0

For peer review only

For peer review only - http://bmjopen.bmj.com/site/about/guidelines.xhtml

4. High/recurrent	753	10.3	3449	4580.3	95	5.7	158	10.3	402	11.9	98	13.9
Covariates												
**Age**												
20 - 24	2252	30.8	2693	1195.6	142	8.5	443	28.8	1412	41.7	255	36.2
25 - 29	3152	43.1	3565	1130.9	959	57.2	693	45.1	1233	36.4	267	37.9
30 - 34	1904	26.1	2212	1161.9	577	34.4	402	26.1	742	21.9	183	26.0
**Language**												
Finnish	6451	88.3	7627	1182.3	1403	83.6	1422	92.5	3020	89.2	606	86.0
Swedish	324	4.4	307	947.5	158	9.4	51	3.3	109	3.2	6	0.9
Other	448	6.1	479	1068.6	40	2.4	64	4.2	252	7.4	92	13.0
**Education**												
Basic education / Lower secondary	3641	49.8	4667	1281.8	273	16.3	232	15.1	2541	75.0	595	84.4
Upper secondary	2319	31.7	2565	1105.9	270	16.1	1211	78.7	755	22.3	83	11.8
Higher education	1348	18.4	1238	918.4	1135	67.6	95	6.2	91	2.7	27	3.8

For peer review only

Table 3. Multinomial logistic regression on occupational class as a determinant of occupational health service (OHS) trajectories among 10,064 City of Helsinki employees aged 20-34 years. Results are based on register data covering the years from 2004 to 2017.

Trajectory	OHS trajectory comparison: Low/increasing vs. No OHS visits	
	Model 1*	Model 2**
**Men**		
Managers or professionals	1.00	1.00
Semi-professionals	1.55 (1.04 - 2.31)	1.31 (0.85 - 2.03)
Routine non-manual workers	1.71 (1.24 - 2.37)	1.40 (0.96 - 2.06)
Manual workers	1.38 (1.00 - 1.90)	1.12 (0.76 - 1.66)
**Women**		
Managers or professionals	1.00	1.00
Semi-professionals	1.25 (1.02 - 1.54)	1.19 (0.92 - 1.53)
Routine non-manual workers	1.47 (1.24 - 1.76)	1.40 (1.11 - 1.76)
Manual workers	1.64 (1.28 - 2.11)	1.55 (1.16 - 2.08)

Trajectory	Low/decreasing vs. No OHS visits	
	Model 1*	Model 2**
**Men**		
Managers or professionals	1.00	1.00
Semi-professionals	1.32 (0.91 - 1.91)	1.45 (0.96 - 2.19)
Routine non-manual workers	1.31 (0.97 - 1.77)	1.53 (1.05 - 2.22)
Manual workers	1.26 (0.94 - 1.68)	1.50 (1.04 - 2.18)
**Women**		
Managers or professionals	1.00	1.00
Semi-professionals	1.48 (1.24 - 1.77)	1.71 (1.36 - 2.15)
Routine non-manual workers	1.33 (1.13 - 1.56)	1.67 (1.34 - 2.07)

For peer review only

Manual workers	1.30 (1.02 - 1.65)	1.65 (1.25 - 2.19)

**Trajectory**	High/recurrent vs. No OHS visits	
	Model 1*	Model 2**
**Men**		
Managers or professionals	1.00	1.00
Semi-professionals	3.21 (1.62 - 6.36)	2.60 (1.23 - 5.49)
Routine non-manual workers	3.99 (2.22 - 7.17)	2.92 (1.48 - 5.74)
Manual workers	5.02 (2.86 - 8.80)	3.56 (1.83 - 6.92)
**Women**		
Managers or professionals	1.00	1.00
Semi-professionals	2.13 (1.61 - 2.81)	2.29 (1.62 - 3.24)
Routine non-manual workers	2.53 (1.98 - 3.25)	2.28 (1.65 - 3.15)
Manual workers	3.10 (2.25 - 4.27)	2.71 (1.85 - 3.97)

Model 1* = Model adjusted for age and first language, Model 2** = M1 + education

For peer review only

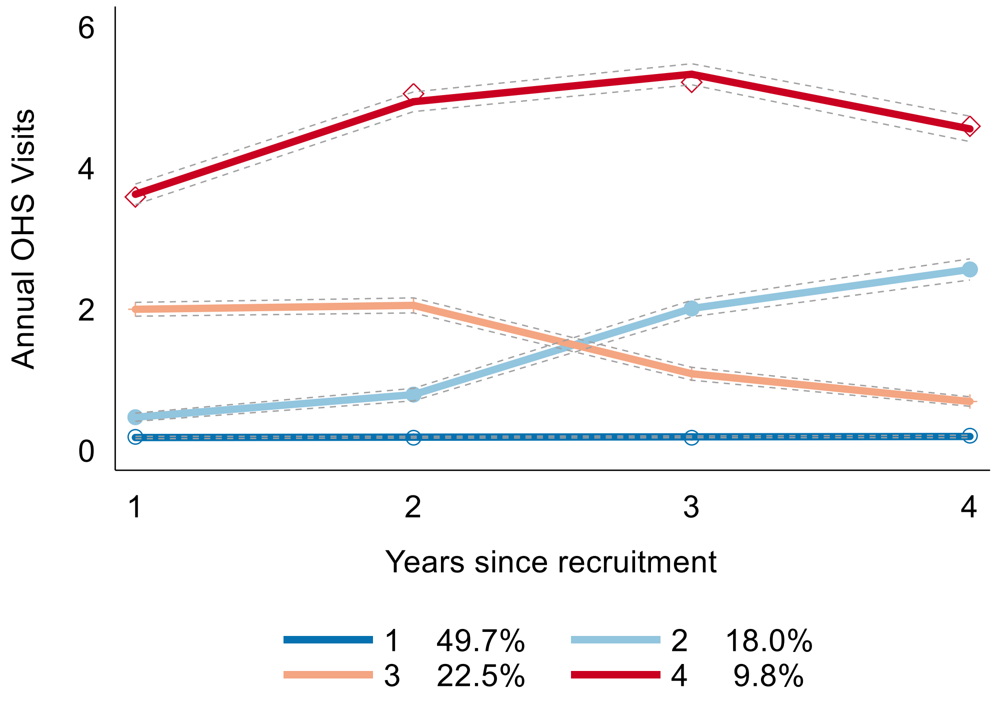

Figure 1. Four occupational health service trajectories, based on registers covering the years 2004 to 2017 among 10,064 the City of Helsinki employees aged 20-34 years. 1 = No visits, 2 = Low/decreasing, 3 = Low/increasing, 4 = High/recurrent use.

STROBE Statement—checklist of items that should be included in reports of observational studies

Young Employees' Trajectories and Occupational Class Differences in Utilization of Primary Care Services Provided by Occupational Health Service

Hilla Sumanen, PhD
Jaakko Harkko, PhD
Kustaa Piha, PhD
Olli Pietiläinen, MSc
Ossi Rahkonen, Professor
Anne Kouvonen, Professor

	Item No	Recommendation	Done, page
**Title and abstract**	1	(*a*) Indicate the study's design with a commonly used term in the title or the abstract	3
		(*b*) Provide in the abstract an informative and balanced summary of what was done and what was found	3
**Introduction**			
Background/rationale	2	Explain the scientific background and rationale for the investigation being reported	4-5
Objectives	3	State specific objectives, including any prespecified hypotheses	5
**Methods**			
Study design	4	Present key elements of study design early in the paper	5
Setting	5	Describe the setting, locations, and relevant dates, including periods of recruitment, exposure, follow-up, and data collection	5-6
Participants	6	(*a*) *Cohort study*—Give the eligibility criteria, and the sources and methods of selection of participants. Describe methods of follow-up<br>*Case-control study*—Give the eligibility criteria, and the sources and methods of case ascertainment and control selection. Give the rationale for the choice of cases and controls<br>*Cross-sectional study*—Give the eligibility criteria, and the sources and methods of selection of participants	5-6
		(*b*) *Cohort study*—For matched studies, give matching criteria and number of exposed and unexposed<br>*Case-control study*—For matched studies, give matching criteria and the number of controls per case	
Variables	7	Clearly define all outcomes, exposures, predictors, potential confounders, and effect modifiers. Give diagnostic criteria, if applicable	6-7
Data sources/ measurement	8*	For each variable of interest, give sources of data and details of methods of assessment (measurement). Describe comparability of assessment methods if there is more than one group	6
Bias	9	Describe any efforts to address potential sources of bias	7, 14-

For peer review only - http://bmjopen.bmj.com/site/about/guidelines.xhtml

			15
Study size	10	Explain how the study size was arrived at	5-6
Quantitative variables	11	Explain how quantitative variables were handled in the analyses. If applicable, describe which groupings were chosen and why	7
Statistical methods	12	(*a*) Describe all statistical methods, including those used to control for confounding	7
		(*b*) Describe any methods used to examine subgroups and interactions	7
		(*c*) Explain how missing data were addressed	
		(*d*) *Cohort study*—If applicable, explain how loss to follow-up was addressed<br>*Case-control study*—If applicable, explain how matching of cases and controls was addressed<br>*Cross-sectional study*—If applicable, describe analytical methods taking account of sampling strategy	
		(*e*) Describe any sensitivity analyses	10

**Results**			**Done, page**
Participants	13*	(a) Report numbers of individuals at each stage of study—eg numbers potentially eligible, examined for eligibility, confirmed eligible, included in the study, completing follow-up, and analysed	5-8
		(b) Give reasons for non-participation at each stage	
		(c) Consider use of a flow diagram	
Descriptive data	14*	(a) Give characteristics of study participants (eg demographic, clinical, social) and information on exposures and potential confounders	7-8
		(b) Indicate number of participants with missing data for each variable of interest	
		(c) *Cohort study*—Summarise follow-up time (eg, average and total amount)	5-6, 8
Outcome data	15*	*Cohort study*—Report numbers of outcome events or summary measures over time	6, 8
		Case-control study—Report numbers in each exposure category, or summary measures of exposure	
		Cross-sectional study—Report numbers of outcome events or summary measures	
Main results	16	(*a*) Give unadjusted estimates and, if applicable, confounder-adjusted estimates and their precision (eg, 95% confidence interval). Make clear which confounders were adjusted for and why they were included	7-10
		(*b*) Report category boundaries when continuous variables were categorized	
		(*c*) If relevant, consider translating estimates of relative risk into absolute risk for a meaningful time period	
Other analyses	17	Report other analyses done—eg analyses of subgroups and interactions, and sensitivity analyses	10
**Discussion**			
Key results	18	Summarise key results with reference to study objectives	10-11
Limitations	19	Discuss limitations of the study, taking into account sources of potential bias or imprecision. Discuss both direction and magnitude of any potential bias	14-15
Interpretation	20	Give a cautious overall interpretation of results considering objectives, limitations, multiplicity of analyses, results from similar studies, and other relevant evidence	10-15
Generalisability	21	Discuss the generalisability (external validity) of the study results	10-15
**Other information**			

For peer review only

Funding	22	Give the source of funding and the role of the funders for the present study and, if applicable, for the original study on which the present article is based	16

*Give information separately for cases and controls in case-control studies and, if applicable, for exposed and unexposed groups in cohort and cross-sectional studies.

Note: An Explanation and Elaboration article discusses each checklist item and gives methodological background and published examples of transparent reporting. The STROBE checklist is best used in conjunction with this article (freely available on the Web sites of PLoS Medicine at http://www.plosmedicine.org/, Annals of Internal Medicine at http://www.annals.org/, and Epidemiology at http://www.epidem.com/). Information on the STROBE Initiative is available at www.strobe-statement.org.

For peer review only

For peer review only - http://bmjopen.bmj.com/site/about/guidelines.xhtml

BMJ Open

Does occupational class explain occupational health service utilization trajectories identified by group-based trajectory analysis among young municipal employees in Finland?

Journal:	*BMJ Open*
Manuscript ID	bmjopen-2018-028742.R1
Article Type:	Original research
Date Submitted by the Author:	14-Aug-2019
Complete List of Authors:	Sumanen, Hilla; South-Eastern Finland University of Applied Sciences, Health care and emergency care; University of Helsinki, Public Health<br>Harkko, Jaakko; University of Helsinki, Social Sciences<br>Piha, Kustaa ; University of Helsinki, Public Health<br>Pietilainen, Olli; University of Helsinki, Department of Public Health<br>Rahkonen, Ossi; University of Helsinki, Department of Public Health<br>Kouvonen, Anne; Helsingin Yliopisto, Faculty of Social Sciences; Queen's University Belfast,
**Primary Subject Heading**:	Health services research
Secondary Subject Heading:	Occupational and environmental medicine
Keywords:	health care visits, socioeconomic differences, young adults

SCHOLARONE™
Manuscripts

Does occupational class explain occupational health service utilization trajectories identified by group-based trajectory analysis among young municipal employees in Finland?

Hilla Sumanen, PhD 1,2,3

Jaakko Harkko, PhD 3

Kustaa Piha, MD, PhD 2

Olli Pietiläinen, MSc 2

Ossi Rahkonen, Professor 2

Anne Kouvonen, Professor 3, 4, 5

1. South-Eastern Finland University of Applied Sciences, Unit of Healthcare and Emergency care and Unit of Research, Development and Innovation
2. University of Helsinki, Faculty of Medicine, Department of Public Health
3. University of Helsinki, Faculty of Social Sciences
4. Research Institute of Psychology, SWPS University of Social Sciences and Humanities
5. Queen's University Belfast, Administrative Data Research Centre – Northern Ireland, Centre for Public Health

Correspondence to: Hilla Sumanen, Department of Public Health, University of Helsinki, Finland, P.O. Box 20 (Tukholmankatu 8B), FIN-00014, University of Helsinki, Finland; hilla.sumanen@helsinki.fi

For peer review only

What is already known about this subject?

This is the first study utilizing longitudinal occupational health service data. All the findings are novel.

What are the new findings?

We identified four occupational health service utilization groups among young employees: "No visits" (50%), "Low/increasing" (18%), "Low/decreasing" (22%) and "High/recurrent" (10%) use. Lower occupational classes had a higher propensity for "High/recurrent" OHS utilization for both genders.

How might this impact on policy or clinical practice in the foreseeable future?

Preventive measures should be targeted particularly to the trajectory groups of "Low/increasing" and "High/recurrent" in order to intervene early. Occupational health service utilization should be more closely monitored among the two lowest occupational classes.

Running head: Young Employees' Utilization of Occupational Health Service

For peer review only

Abstract

OBJECTIVES: To identify groups of municipal employees between the ages of 20 and 34 years with distinct utilization trajectories of primary care services provided by occupational health service (OHS), measured as the annual number of OHS visits, and to identify demographic and socio-economic risk factors that distinguish employees in the high utilization trajectory group(s).

METHODS: The present study is a retrospective register-based cohort study. All municipal employees of the City of Helsinki, Finland, aged 20-34 in the Helsinki Health Study (HHS), recruited from 2004 to 2013, with follow-up data for four years were included in the study (n = 9762). The outcome measure was group-based trajectories of OHS utilization, identified with a group-based trajectory analysis (GBTA). The demographic and socio-economic variables used to predict the outcome were age, first language, educational level and occupational class. The analyses were stratified by gender.

RESULTS: A large proportion of the young employees do not utilize OHS. Trajectory groups of “No visits” (50%), “Low/increasing” (18%), “Low/decreasing” (22%) and ”High/recurrent” (10%) use were identified. We found occupational class differences in OHS utilization patterns showing that lower occupational classes had a higher propensity for “High/recurrent” OHS utilization for both genders.

CONCLUSIONS: Preventive measures should be targeted particularly to the trajectory groups of “Low/increasing” and “High/recurrent” in order to intervene early. In addition, OHS utilization should be closely monitored among the two lowest occupational classes. More research with longitudinal OHS data is needed.

Key terms: Health care visits, Socioeconomic differences, Young adults, Women, Men

Strengths and limitations of this study

- This is the first study utilizing longitudinal occupational health service data
- The sample consisted all of 20-34-year-old employees of the City of Helsinki. The occupational health care policies are same for all these employees and have remained same during the study period of 2004-2017.
- Our study avoids a common limitation of previous occupational health service studies that are based on limited samples with data on only those attending the service or those responding to survey.
- Limitations of the data include the lack of diagnostic information and lifestyle factors.
- The lack of information about other primary care visits outside the occupational health service further limits the interpretation of the results.

For peer review only

Introduction

Finland has a unique occupational health service (OHS) system with statutory prevention of occupational health hazards (preventive services) and additionally purchased primary care services. OHS may be provided by employer's own OHS units, private clinics or public sector health centers with specifically trained occupational health physicians, nurses, physiotherapists and psychologists. In Finland, most employers purchase the additional primary care element for their employees. In 2017, 94% of the employees covered by statutory preventive OHS also had an access to primary care for any illness including all non-work related illnesses, paid by their employer and partly subsidized by the National Pension Fund [1]. OHSs are free for employees at the point of delivery and their accessibility is typically good, making them the main source and the preferred type of primary care for Finnish employees.

The utilization of OHS in Finland or elsewhere has been only scarcely studied, and especially studies using longitudinal data are lacking. There are few previous Finnish studies concentrating on the OHS primary care visits with cross-sectional study designs and from the viewpoint of service utilization over the course of 6 to 12 months. A recent study [2] with data from a large private Finnish OHS provider investigated the top 10% frequent attenders in primary care services in 2015. The results showed that frequent attendance was associated with female gender, being employed by a medium or a large company, working in the manufacturing industry, public administration or in health and social care services. In an earlier study with survey data (N=1636) from the Finnish working-age population [3], 57% of Finnish employees covered by the OHS primary care visited either their occupational health physician or nurse due to any illness during the 6-month period. In that study, those visits were strongly associated with chronic illness impacting occupational health and work ability. Both previous studies utilizing Finnish OHS data acknowledge the lack of research focusing to these unique services provided for the working population and identify the need for further study to identify service development needs and possibilities.

For peer review only

The current study utilizes longitudinal data from the own OHS unit of Finland's largest employer. The City of Helsinki offers same OHSs with primary care for all its employees (n=~38 000 per year) with no cost for the employees. The City of Helsinki employees have been the focus of the Helsinki Health Study [4] since 2000, but this is the first study using their OHS data. Our focus is on the younger employees and their OHS utilization, as previous studies have shown that they have a high prevalence of sickness absence (SA) [5] and there are already large socioeconomic differences in SA apparent in the younger age groups [6-7]. Reducing SA is high on employers' agenda. Service utilization information is important for planning preventive actions via targeted interventions or improving case management protocols [8-9] arranged by the OHS. According to extensive Finnish and international evidence socioeconomic differences in SA are large among employees [10-21], thus the differences in OHS utilization is feasible to monitor from the socioeconomic viewpoint and to be able to identify groups for interventions.

In our study, we aimed to identify developmental trajectories of OHS primary care service utilization among 20-34-year-old municipal employees of the City of Helsinki. In the second stage of the analysis we aimed to identify occupational class differences in belonging to different trajectory groups. We tested two hypothesis, first, that the distinct trajectories can be identified, and second, that occupational class gradient can be found in OHS utilization.

For peer review only

Methods

This is a retrospective register-based cohort study. The study is a part of the Helsinki Health Study on health and well-being among employees of the City of Helsinki, Finland [4]. The study included all Helsinki City employees aged 20-34 at the beginning of their first work contract with the City (N = 22,576) between 04/06/2004 and 04/19/2013. The selection of this age group was based on the Eurostat definition of young employees ,and on previous studies investigating the occurrence of

illness and sickness absence in employees of different ages [22, 23]. For each employee the follow-up started from their initial recruitment. Employees with incomplete data on occupational position (n= 754) and those with employment record for less than four years (n= 12,512) were excluded from the present study and we ended up with a sample size of n = 9,762. We excluded employees with less than four years of employment history as we needed a long enough follow-up time to observe potential development trajectories. The descriptive characteristics of excluded subjects are found in web-appendixes 1 and 2. The length of the follow-up was measured as calendar days in employment.

OHS utilization trajectory was the outcome of the study. The trajectory, i.e. the developmental course of OHS utilization, was measured from four consecutive data points indicating annual number of outpatient primary care visits for each employee. The number of visits ranged from 0 to 103. The demographic and socioeconomic variables used to predict the outcome were age, first language, educational level and occupational class. The analyses were stratified by gender.

We used four occupational class groups. Based on the socioeconomic classification of Statistics Finland and the occupational classification of the City of Helsinki [4,12], non-manual employees were divided into three groups based on skills requirements and supervisory status: Managers and professionals, semi-professionals, and routine non-manual employees. Managers have subordinates and they do managerial or administrative work, whereas professionals include employees with a university degree, such as physicians and teachers. Semi-professionals include occupations such as registered nurses and technicians. Routine non-manual employees include clerical employees and lesser-educated occupations particularly within the social and health care, such as child-minders and care workers. The fourth occupational class, manual workers, include occupations for example from the fields of cleaning, kitchen work and public transport.

Age was categorized into three groups: 20-24, 25-29 and 30-34-year-olds. First language was categorized as: Finnish, Swedish, Other. Education was classified into three levels: higher education

For peer review only

(a Master's or a doctor's degree), upper secondary (a Bachelor's degree), and lower secondary (upper-secondary school, vocational school) or basic education (comprehensive school).

The employer's personnel and occupational health care registers were used to obtain socio-demographic characteristics and information on OHS use. Educational level was obtained from annually updated Statistics Finland's registry of completed education and degrees, and was linked to the City of Helsinki personnel register.

Ethics

The study follows the Helsinki Health Study protocol in line with the University of Helsinki's guidelines and data legislation. The ethics committees of the Department of Public Health, the University of Helsinki and the health authorities of the City of Helsinki have approved the HHS study. The City of Helsinki has given permission for data linkage.

Patient and Public Involvement statement

No Patient or Public Involvement.

Statistical methods

Group-based trajectory modeling (GBTM) with Stata's *traj* command [24] was applied to identify clusters of individuals, or trajectory groups, with similar developmental trajectory on OHS utilization. The method assigns a subject to a trajectory group by assessing the probability of group membership. The count variables were assumed to be Poisson-distributed and zero inflated Poisson models were applied. The ideal number of trajectory groups and trajectory shapes were assessed by four criteria suggested by the existing literature: Bayesian information criteria (BIC), posterior probabilities of trajectory group membership higher than .70, sizes of trajectory groups larger than 5% and a distinct interpretability of the identified trajectory groups [25,26]. Subsequently,

For peer review only

multinomial logistic regression models using Stata's *mlogit* command were applied to investigate the role of occupational class as a predictor of the trajectory group membership. In a two-step-analysis, estimates are given for occupational class, first, adjusted for age and first language and, second, additionally adjusted for education. The trajectory cluster indicating the lowest health care utilization was defined as the reference group in the analysis. The results are given as relative risk ratios (RRRs) with their 95% confidence intervals (CIs). All statistical analyses were performed with Stata 15.

Results

Descriptive results

The study sample included 9762 the City of Helsinki employees aged between 20 and 34 at the beginning of the follow-up. 27% of the employees were men and 73% women. Among men, the yearly average of OHS visits was 1.03 (SD, 1.44) during the mean of 1341 days of follow-up. Among women, the yearly average was 1.16 (SD, 1.50) OHS visits in the mean of 1328 days. Of the subjects of the study, 2272 (23%) were managers or professionals, 1824 (19%) semi-professionals, 4064 (42%) routine non-manual workers, and 1602 (16%) manual workers (Tables 1-2). Managers/professionals had an equal gender distribution, semi-professionals and routine non-manual workers were more often women, whereas men constituted the majority of manual workers. Occupational class was closely linked to educational attainment in both genders.

In trajectory analysis, a trajectory model consisting of four distinct trajectories including 1 trajectory with a linear, 1 with a quadratic, and 2 with a cubic shape showed the best fit using the BIC criterion (Figure 1). The largest identified trajectory group "No visits" (n = 5,106, 50%) represents those with less than 0.2 annual OHS visits over the four years of follow-up. There were two intermediate groups. The group labeled "Low/increasing" (n = 1,744, 18%) is characterized by

For peer review only

low number of visits during the first two years followed by a slight increase in visits during the next two years. The group “Low/decreasing” (n = 2,238, 22%) follows a similar pattern as the group 2, but in a reverse order. The members of the both low groups averaged 1.5 annual OHS visits during the follow-up. The “High/recurrent” group (n = 976, 10%) consists of employees characterized by high levels of OHS visits, with an average of 4.6 visits per year, from the start of the employment to the end of the follow-up. The mean assignment probabilities were 0.93 for the "No visits", 0.81 for the "Low/increasing", 0.82 for the "Low/decreasing”, and 0.92 for the "High/recurrent" trajectory groups, indicating a good model fit to the data. Of men, 54% belonged to the “No visits” trajectory and 8.4% to the “High/recurrent” trajectory group, whereas the corresponding figures for women were 50% and 10%, indicating a higher propensity for women to belong to the “High/recurrent” trajectory group. The assignment of the members of different occupational classes to different OHS trajectories followed the socio-economic gradient. Of managers or professionals, 59% belonged to the “No visits” trajectory and 5% to the “High/recurrent” trajectory. The corresponding figures for semi-professionals were 48% and 10%, for routine non-manual workers 48% and 11%, and for manual workers 49% and 13%, respectively.

*** Figure 1 ***

*** Table 1 ***

*** Table 2 ***

OHS utilization trajectories by occupational class

Occupational class was a strong independent predictor for the OHS utilization trajectories, as demonstrated in Table 3. The likelihood of belonging to the “High/recurrent” trajectory was

For peer review only

increased for those being in a lower occupational class. For both women and men, the risk for belonging to the “High/recurrent” trajectory was highest for manual workers, followed by routine non-manual workers and semi-professionals.

The independent effect of occupational class remained after adjustment for all covariates including age, first language and education. The association was most evident in the “High/recurrent” trajectory. The relative risk for this group membership was 2.92 (95% CI, 1.48 – 5.74) for male routine non-manual workers and 3.56 (95% CI, 1.83 – 6.92) for male manual workers. The corresponding figures for women were 2.28 (95% CI, 1.65 – 3.15) and 2.71 (95% CI, 1.85 – 3.97), respectively. The results indicate that a proportion of the association between occupational class and belonging to the “High/recurrent” trajectory is dependent on the lower educational attainment of the members of the lower occupational classes. The results comparing the two low trajectories with the “No visits” OHS trajectory were less clear in terms of statistical significance. Whereas all estimates but one in “Low/decreasing” vs “No visits” comparison remained statistically significant after full adjustment, four out of “Low/increasing” vs “No visits” comparisons become statistically non-significant in the final model. The observed excess risks generated by occupational class were thus smaller in both “Low” trajectories compared to “High/recurrent” trajectory. Notably, the relative risks related to the membership of these middle trajectories were not manifested in a dose-exposure manner as was the case with the “High/recurrent” trajectory.

*** Table 3 ***

Sensitivity analyses

To assess the extent the parameter estimates were sensitive to potential errors in model specification and data, three types of sensitivity analyses were performed. First, we reproduced estimates from

For peer review only

the original data with bootstrap resampling (1000 replications). Second, we reproduced the results with logistic regression analyses defining the high-utilization group as those whose total number of OHS visits during the follow-up period was 10 or more (n=1,484) was compared with those with no or just one visit (n= 3,993). These sensitivity analyses indicated robustness of our inference about the relationship between occupational class and OHS utilization trajectories. Third, we performed a sensitivity analysis where variables on part-time work and fixed-term contract were included, but they changed the estimates only modestly.

Discussion

In this study we identified developmental trajectories and socioeconomic differences in OHS primary care service utilization among 20 to 34-year-old employees of the City of Helsinki from 2004 to 2017. Our key results were: 1) Half of the young employees did not use occupational health care services to any considerable extent; 2) Higher occupational classes utilized less OHS; 3) Four trajectory groups, that is, “No visits”, ”Low/increasing”, “Low/decreasing” and “High/recurrent”, were identified. 4) The trajectory group of “High/recurrent” included a larger number of lower class workers especially among men, and the differences were large also among women. 5) Occupational class differences in “Low/decreasing” group were evident in both genders. 6) Only in women there were some occupational class differences in belonging to the trajectory group of “Low-increasing OHS utilization”.

Our results highlight the significance of socioeconomic gradient in OHS utilization that was visible both in men and women. The percentage of those who had no visits was the highest among managers and professionals and the proportion of no visits decreased when going down the occupational class ladder. Respectively, high and recurrent use was smallest among managers and professionals, and increased with decreasing occupational class, this type of use being the most

For peer review only

common among manual workers. A larger proportion of men had no visits at all in each occupational class, thus the women used the health services more, in line with earlier findings [2]. In a similar way, the high and recurrent use was higher among women than among men. In the present context, primary care visits can be interpreted as an indicator of incidence of acute illnesses, as the Finnish OHS system distinguish visits related to occupational health hazards. The present results concerning primary care visits are in line with previous findings from our own and other studies showing the socioeconomic differences in sickness absence among employees and the gender differences in sickness absence, i.e. women having more absence than men [7,10,14,18,21]. It can be assumed that large number of OHS visits precede sickness absence as also indicated by Bergh et al. [27].

Stratified analyses indicated gender differences in OHS utilization. According to our results, among men the occupational class differences disappeared after full adjustment in the trajectory group of "Low/increasing". This implies that the initial differences are associated with the type of work tasks. In contrast, after full adjustment, among women the employees in the two lowest occupational classes had a higher risk for belonging to this trajectory group. In the trajectory group of "Low/decreasing" the differences were initially similar among the three lowest occupational classes. However, the differences modestly increased after full adjustment, implying that there are several factors associated with the low OHS utilization. This was seen among both genders.

The trajectory group of "High/recurrent" is perhaps the most interesting group alongside with the "Low/increasing" group in terms of costs and possible preventive opportunities. According to our results the occupational class differences in this group are steep especially among men, and also large among women. After full adjustment the differences decreased more in men, suggesting that the initial differences are more associated with work tasks among them. However, the differences remained high in both genders after adjustments. Studies regarding socioeconomic differences in

For peer review only

sickness absence have also found that the differences are steeper among men [7,17,18], but the former studies mostly concentrate on older employees.

The differences in physical and psychosocial demands between occupational classes are important to take into account when interpreting the results. Manual workers have more physically demanding jobs, which may affect their need for primary care services. Adverse working conditions may cause ill-health and need of health services as milder even health difficulties may prevent these employees from working. Employees in higher occupational classes typically have more complex and mentally demanding jobs [28]. In studies examining the socioeconomic differences in sickness absence, physical working conditions have been found to be the strongest explanatory factor [12,16]. However, employees in our study are fairly young and thus adverse physical or psychosocial working conditions might not have yet affected their health, as health-related effects usually increase with age. In addition, the unique OHS system where the visits associated with occupational health hazards (preventive services) are recorded separately from primary care visits may contribute to the differences seen in our results. For example visits with more chronic work-related reason are usually not recorded as primary care visits. Thus, the overall utilization of OHS requires further research.

Our study indicates that service use patterns might recognize vulnerable groups more precisely than just belonging to certain occupation or occupational class may do. Despite this, the two lowest occupational classes may need extra attention based on their OHS utilization patterns. Case management protocols are essential in coordinating patient-centered care path which also saves costs [8,9,29]. Among younger employees, timely treatment is highly important, as it might prevent the worsening of their condition. OHS should identify those employees who use services a lot.

Previous longitudinal studies using OHS data are lacking, but recent studies have showed that frequent utilization of OHS was associated with psychiatric problems and musculoskeletal disorders [2], whereas the latter also predicts persistent frequent utilization [30]. Furthermore, frequent

For peer review only

utilization has increased the risk of long sickness absence [31] and disability pension [32]. These associations highlight the need of identifying those in risk for more severe illness and work disability at an early stage, and information on the different utilization trajectories with identified occupational class differences supports these preventive actions. Moreover, some comparisons can be made with the studies investigating frequent attenders in primary care. Frequent attenders in primary care in the general population have been studied particularly in Netherlands and in Sweden using questionnaire surveys and record linkage. These studies had participants from a wide range of sociodemographic backgrounds and they consider only visits to general practitioners, thus their direct comparability to OHS utilization is difficult. In addition, the definition of frequent attender varies between studies [33]. However, previous studies have found out that frequent attenders have multiple reasons for presenting [34], but overall chronic illnesses [33], somatic diseases and symptoms [35,36] and especially psychiatric problems [35] have been associated with more frequent primary care service use. Frequent attenders have more health discomfort, low mastery, and they may be more vulnerable for stressful life events due to inadequate coping strategies [27,37,38]. In line with the study by Reho et al. [31,32], frequent attenders are a high-risk group for long-term sickness absence and disability pension also in general population [27]. According to two previous Dutch studies one out of every seven 1-year frequent attenders becomes persistent frequent attender and six out of seven are transient frequent attenders [35,39]. Based on this previous evidence the inclusion of diagnostic information would be important in future studies of OHS utilization. However, from the methodological viewpoint both the Dutch and Swedish research groups point out that age should be taken into account when studying the frequent attenders, as not only the reasons for high service utilization but also what constitutes high use highly differ by age group [36,40].

For peer review only

Methodological considerations

The registers used in this study are reliable and comprehensive. We focused on all occupational groups within the far largest municipality in Finland and the sample consisted all of 20-34-year-old employees within this organization. The occupational health care policies are same for all these employees and have remained same during the study period of 2004-2017. Our study avoids a common limitation of OHS studies that they are based on limited samples with data on only those attending the service (e.g. [2]) or those responding to survey [3]. Another advantage of this study is that we could make inferences based on longitudinal cohort data instead of relying on cross-sectional evidence. Limitations of the data include the lack of information of diagnoses, physical and psychosocial working conditions, lifestyle factors and any primary care visits outside the OHS. In addition, people with initial poor health may attain lower educational level and end up in lower occupational positions. Unfortunately, our data does not extend beyond the current employment relations.

The OHS system in Finland is unique, thus comparison to other countries is difficult. The principle of primary care use is similar as in the general practice (GP) or family doctor setting in most other Western countries, but the patient population differs to some extent from ours in terms of demographics and employment status. In Finland, the employer offers (most employers do) those services and thus OHS is the main source for primary care for employees due to being free at the point of delivery and enabling an easy access. However, even within Finland, different employers can have different policies in terms of provision of primary care services. Nevertheless, our results can be broadly generalized to Finnish public sector employees.

The present study is to our knowledge the first one that used longitudinal latent class analysis aiming to capture OHS utilization as a complex longitudinal phenomenon. GBTM approach mixes the application of formal statistical criteria and subjective evaluation in model fitting [24]. One of its strength is that it allowed us to identify high OHS utilization over time. It is a limitation that

For peer review only

those who left the City of Helsinki within the first four years of their employment were lost to follow-up. Another benefit is that GBTM is capable of identifying different OHS trajectories within subjects that appear similar in terms of summary statistics. In this study we were able to distinguish between two “low” trajectories, which may allow for better planning of targeted prevention measures. However, we want to highlight that by this methodology it cannot be ascertained that the observed subgroups are distinct population subgroups. As in case of any latent trajectory class analyses, there is a possibility that the data could be interpreted as homogenous but non-normal [41]. We find, however, the obtained groups to be realistic and the results applicable in terms of real-life interpretations. However, further analysis with longer follow-up would be important to confirm the trajectories found.

Conclusions

We used GBTM for distinguishing four different developmental trajectories in OHS primary care service utilization among 20-34-year-old employees of the City of Helsinki. Occupational class differences exist in the utilization development trajectories. A large proportion of the young employees do not use OHS primary care services and non-use is most common among the highest occupational class. Especially trajectories where the utilization has grown or been high throughout the follow-up had large occupational class differences, which followed the socioeconomic gradient. Identifying high utilization patterns is important as ten per cent of employees that may be labeled high and recurrent users account for 40% of the all OHS consultations.

According to our results preventive measures should be considered among the trajectory groups of “Low/increasing” and “High/recurrent”. In addition, attention should be paid to the two lowest occupational classes, and their OHS utilization should be closely monitored by the occupational

For peer review only

health care in order to identify those in need for extra support. OHS utilization requires more longitudinal research. Case management protocols should be further developed.

Author contributions

HS, AK & JH designed the study. HS mainly wrote the manuscript with contributions and comments from all the other authors (JH, KP, OP, OR & AK), JH did the statistical analyses and wrote the results section and OP commented those. All the authors have approved the final version of the manuscript.

Conflicts of Interest

Authors declare no conflicts of interest.

Funding

This study was funded by the Finnish Work Environment Fund (grant 117321) and the Academy of Finland (grant 315343). AK was funded by the Economic and Social Research Council (ESRC) (grant ES/L007509/1 and ES/S00744X/1). OR was funded by the Academy of Finland (grant 1294514). The funders had no involvement to the preparation of the manuscript.

Data sharing statement

Data cannot be shared publicly because it contains confidential medical information and the study participants have not given their permission to data sharing. Data are kept at the University of Helsinki computers and are available upon agreement with the Helsinki Health Study for researchers who meet the criteria for access to confidential data.

For peer review only

References

1. Kelasto-reports. 2019. Kelan työterveyshuoltotilasto 2017 (in Finnish). Official statistics of Finland. https://helda.helsinki.fi/bitstream/handle/10138/303534/ Kelan_tyoterveyshuoltotilasto_2017.pdf?sequence=1&isAllowed=y. (Accessed 6 Aug 2019).

2. Reho T, Atkins S, Talola N, Viljamaa M, Sumanen M, Uitti J. Frequent attenders in occupational health primary care: A cross-sectional study. Scand J Public Health 2018;1:1403494818777436.

3. Kimanen A, Rautio M, Manninen P, Räsänen K, Husman P, Husman K. Primary care visits to occupational health physicians and nurses in Finland. Scand J Public Health 2011;39:525-32.

4. Lahelma E, Aittomäki A, Laaksonen M, Lallukka T, Martikainen P, Piha K, et al. Cohort Profile: The Helsinki Health Study. Int J Epidemiol 2013;42:722–30.

5. Sumanen H, Pietiläinen O, Lahti J, Lahelma E, Rahkonen O. Sickness absence among young employees: trends from 2002 to 2013. J Occup Health 2015;57:474-81.

6. Sumanen H, Lahelma E, Lahti J, Pietiläinen O, Rahkonen O. Educational differences in sickness absence trends among young employees from 2002 to 2013 in Helsinki, Finland. BMJ Open 2016; 6:e008550.

7. Sumanen H, Lahelma E, Pietiläinen O, Rahkonen O. The Magnitude of Occupational Class Differences in Sickness Absence: 15-Year Trends among Young and Middle-Aged Municipal Employees. Int J Environ Res Public Health 2017;14:E625.

8. AAOHN. Case Management: The Occupational and Environmental Health Nurse Role. Workplace Health Saf 2013;61:8.

9. Robinson M, Fisher TF, Broussard K. Role of Occupational Therapy in Case Management and Care Coordination for Clients With Complex Conditions. Am J Occup Ther 2016;70:7002090010p1-7002090010p6.

For peer review only

10. Piha K, Laaksonen M, Martikainen P, Rahkonen O, Lahelma E. Interrelationships between education, occupational class, income and sickness absence. Eur J Public Health 2010;20:276-80.

11. Piha K, Martikainen P, Rahkonen O, Roos E, Lahelma E. Trends in socioeconomic differences in sickness absence among Finnish municipal employees 1990-99. Scand J Public Health 2007;35:348-55.

12. Laaksonen M, Piha K, Rahkonen O, Martikainen P, Lahelma E. Explaining occupational class differences in sickness absence: Results from middle-aged municipal employees. J Epidemiol Community Health 2010;64:802–7.

13. Sumanen H, Pietiläinen O, Lahti J, Lahelma E, Rahkonen O. Interrelationships between education, occupational class and income as determinants of sickness absence among young employees in 2002-2007 and 2008-2013. BMC Public Health 2015;15:332.

14. Pekkala J, Blomgren J, Pietiläinen O, Lahelma E, Rahkonen O. Occupational class differences in diagnostic-spesific sickness absence: A register-based study in the Finnish population. BMC Public Health 2017;17:670.

15. Feeney A, North F, Head J, Canner R, Marmot M. Socioeconomic and sex differentials in reason for sickness absence from the Whitehall II study. Occup Environ Med 1998;55:91-8.

16. Christensen K, Labriola M, Lund T, Kivimäki M. Explaining the social gradient in long-term sickness absence: A prospective study of Danish employees. J Epidem Community Health 2008;62:181–3.

17. Hansen H, Ingebrigtsen T. Social class and sickness absence in Norway. Acta Sociologica 2008;51:309–27.

18. Löve J, Hensing G, Holmgren K, Toren K. Explaining the social gradient in sickness absence: A study of a general working population in Sweden. BMC Public Health 2013;13:545-2458-13-545.

For peer review only

19. Vahtera J, Virtanen P, Kivimäki M, Pentti J. Workplace as an origin of health inequalities. J Epidemiol Community Health 1999;53:399-407.

20. Morikawa Y, Martikainen P, Head J, Marmot M, Ishizaki M, Nakagawa H. A comparison of socio-economic differences in long-term sickness absence in a Japanese cohort and a British cohort of employed men. Eur J Public Health 2004;14:413-6.

21. Kaikkonen R, Härkänen T, Rahkonen O, Gould R, Koskinen S. Explaining educational differences in sickness absence: a population-based follow-up study. Scand J Work Environ Health 2015;41:338-46.

22. Sumanen H. Work disability among young employees: Changes over time and socioeconomic differences. Dissertationes Scholae Doctoralis Ad Sanitatem Investigandam Universitatis Helsinkiensis; nro 18/2016.

23. Eurostat. 2019. Participation of Young People in Education and the Labour Market. https://ec.europa.eu/eurostat/statistics-explained/index.php?title=Participation_of_young_people_in_education_and_the_labour_market&oldid=433402#Participation_of_young_persons_in_formal_education_and_in_the_labour_market. (Accessed 6 Aug 2019)

24. Jones BL, Nagin DS. 2012. A Stata Plugin for Estimating Group-Based Trajectory Models. https://ssrc.indiana.edu/doc/wimdocs/2013-03-29_nagin_trajectory_stata-plugin-info.pdf (accessed 1 Jun 2018)

25. Nagin D. 2005. Group-Based Modeling of Development. Cambridge, Mass: Harvard University Press.

26. Nagin DS, Odgers CL. Group-Based Trajectory Modeling in Clinical Research. Annu Rev Clin Psychol 2010;6:109–38.

For peer review only

27. Bergh H, Baigi A, Månsson J, Mattsson B, Marklund B. Predictive factors for long-term sick leave and disability pension among frequent and normal attenders in primary health care over 5 years. Public Health 2007;121:25-33.

28. Nieuwenhuijsen K, Verbeek JH, de Boer AG, Blonk RW, van Dijk FJ: Predicting the duration of sickness absence for patients with common mental disorders in occupational health care. Scand J Work Environ Health 2006;32:67-74.

29. Golden R. The importance of collaboration. CMSA Today 2015;4:21-2.

30. Reho T, Atkins S, Talola N, Sumanen M, Viljamaa M, Uitti J. Comparing occasional and persistent frequent attenders in occupational health primary care - a longitudinal study. BMC Public Health 2018;26:1291.

31. Reho TTM, Atkins SA, Talola N, Sumanen MPT, Viljamaa M, Uitti J. Occasional and persistent frequent attenders and sickness absences in occupational health primary care: a longitudinal study in Finland. BMJ Open 2019;19:e024980.

32. Reho TTM, Atkins SA, Talola N, Sumanen MPT, Viljamaa M, Uitti J. Frequent attenders at risk of disability pension: a longitudinal study combining routine and register data. Scand J Public Health 2019;11:1403494819838663.

33. Vedsted P, Christensen MB. Frequent attenders in general practice care: a literature review with special reference to methodological considerations. Public Health 2005;119:118-37.

34. Smits FT, Brouwer HJ, Schene AH, van Weert HC, Ter Riet G. Is frequent attendance of longer duration related to less transient episodes of care? A retrospective analysis of transient and chronic episodes of care. BMJ Open 2016;6:e012563.

For peer review only

35. Smits FT, Brouwer HJ, ter Riet G, van Weert HC. Epidemiology of frequent attenders: a 3-year historic cohort study comparing attendance, morbidity and prescriptions of one-year and persistent frequent attenders. BMC Public Health 2009:9;36.

36. Bergh H, Marklund B. Characteristics of frequent attenders in different age and sex groups in primary health care. Scand J Prim Health Care 2003;21:171-7.

37. Bergh H, Baigi A, Fridlund B, Marklund B. Life events, social support and sense of coherence among frequent attenders in primary health care. Public Health 2006;120:229-36.

38. Smits FT, Brouwer HJ, Zwinderman AH, Mohrs J, Schene AH, van Weert HC, ter Riet G. Why do they keep coming back? Psychosocial etiology of persistence of frequent attendance in primary care: a prospective cohort study. J Psychosom Res 2014;77:492-503.

39. Smits FT, Brouwer HJ, van Weert HC, Schene AH, ter Riet G. Predictability of persistent frequent attendance: a historic 3-year cohort study. Br J Gen Pract 2009;59:e44-50.

40. Smits FT, Mohrs JJ, Beem EE, Bindels PJ, van Weert HC. Defining frequent attendance in general practice. BMC Fam Pract.2008;15:21.

41. Bauer DJ, Curran PJ. Distributional Assumptions of Growth Mixture Models: Implications for Overextraction of Latent Trajectory Classes. Psychological Methods 2003;8:338-63.

For peer review only

Table 1. Descriptive statistics of the four occupational classes among 2,454 male The City of Helsinki employees aged 20-34 years. Results are based on register data covering the years from 2004 to 2017.

	**Total**				**Occupational class**							
			**OHS visits p.a.**		*Managers or professionals*		*Semi-professionals*		*Routine non-manual workers*		*Manual workers*	
	N	% / (sd.)	N	1 / 1 000	*n*	*% / (sd.)*	*n*	*% / (sd.)*	*n*	*% / (sd.)*	*n*	*% / (sd.)*
**Total**	2454	100.0	2528	1030.3	594	100.0	286	100.0	677	100.0	897	100.0
The length of the follow-up in days, average (sd.)	1341	(252)			1371	(204)	1382	(191)	1290	(292)	1348	(260)
OHS visits per annum, 1 / 1000 (sd.)	1030.3	(1435.3)			705.4	(939.4)	996.5	(1151.3)	1121.5	(1482.1)	1187.3	(1695.8)
**Outcome: Trajectory group**												
1. No OHS visits	1337	54.5	225	168.5	374	63.0	148	51.7	349	51.6	466	52.0
2. Low/increasing	420	17.1	626	1489.3	91	15.3	54	18.9	131	19.4	144	16.1
3. Low/decreasing	496	20.2	728	1468.2	113	19.0	63	22.0	138	20.4	182	20.3
4. High/recurrent	201	8.2	949	4722.6	16	2.7	21	7.3	59	8.7	105	11.7
Covariates												
**Age (years)**												
20 - 24	618	25.2	684	1107.2	23	3.9	38	13.3	221	32.6	336	37.5
25 - 29	1077	43.9	996	924.6	299	50.3	152	53.1	296	43.7	330	36.8
30 - 34	759	30.9	848	1117.6	272	45.8	96	33.6	160	23.6	231	25.8
**First language**												
Finnish	2154	87.8	2219	1029.9	499	84.0	272	95.1	589	87.0	794	88.5

For peer review only

Swedish	81	3.3	36	438.3	47	7.9	2	0.7	24	3.5	8	0.9
Other	193	7.9	256	1327.7	24	4.0	12	4.2	62	9.2	95	10.6
**Education**												
Basic education / Lower secondary	1595	65.0	1807	1132.8	136	22.9	115	40.2	530	78.3	814	90.7
Upper secondary	476	19.4	453	951.2	126	21.2	149	52.1	130	19.2	71	7.9
Higher education	383	15.6	269	701.7	332	55.9	22	7.7	17	2.5	12	1.3

Table 2. Descriptive statistics of the four occupational classes among 7,308 female The City of Helsinki employees aged 20-34 years. Results are based on register data covering the years from 2004 to 2017.

	Total				Occupational class							
			OHS visits p.a.		*Managers or professionals*		*Semi-professionals*		*Routine non-manual workers*		*Manual workers*	
	N	% / (sd.)	N	1 / 1 000	*n*	*% / (sd.)*	*n*	*% / (sd.)*	*n*	*% / (sd.)*	*n*	*% / (sd.)*
**Total**	7308	100.0	8470	1158.9	1678	100.0	1538	100.0	3387	100.0	705	100.0
The length of the follow-up in days, average (sd.)	1328	(254)			1326	(244)	1375	(202)	1310	(272)	1322	(281)
OHS visits per annum, 1 / 1000 (sd.)	1158.9	(1499.2)			850.9	(1114.0)	1141.7	(1357.4)	1268.7	(1627.4)	1402.5	(1808.0)
**Outcome: Trajectory group**												
1. No OHS visits	3623	49.6	644	177.7	972	57.9	734	47.7	1599	47.2	318	45.1
2. Low/increasing	1281	17.5	1922	1500.2	264	15.7	249	16.2	627	18.5	141	20.0
3. Low/decreasing	1651	22.6	2455	1487.0	347	20.7	397	25.8	759	22.4	148	21.0

For peer review only

4. High/recurrent	753	10.3	3449	4580.3	95	5.7	158	10.3	402	11.9	98	13.9
Covariates												
**Age**												
20 - 24	2252	30.8	2693	1195.6	142	8.5	443	28.8	1412	41.7	255	36.2
25 - 29	3152	43.1	3565	1130.9	959	57.2	693	45.1	1233	36.4	267	37.9
30 - 34	1904	26.1	2212	1161.9	577	34.4	402	26.1	742	21.9	183	26.0
**Language**												
Finnish	6451	88.3	7627	1182.3	1403	83.6	1422	92.5	3020	89.2	606	86.0
Swedish	324	4.4	307	947.5	158	9.4	51	3.3	109	3.2	6	0.9
Other	448	6.1	479	1068.6	40	2.4	64	4.2	252	7.4	92	13.0
**Education**												
Basic education / Lower secondary	3641	49.8	4667	1281.8	273	16.3	232	15.1	2541	75.0	595	84.4
Upper secondary	2319	31.7	2565	1105.9	270	16.1	1211	78.7	755	22.3	83	11.8
Higher education	1348	18.4	1238	918.4	1135	67.6	95	6.2	91	2.7	27	3.8

For peer review only

Table 3. Multinomial logistic regression on occupational class as a determinant of occupational health service (OHS) trajectories among 9762 City of Helsinki employees aged 20-34 years. Results are based on register data covering the years from 2004 to 2017.

	OHS trajectory comparison	
**Trajectory**	Low/increasing vs. No OHS visits	
	Model 1*	Model 2**
**Men**		
Managers or professionals	1.00	1.00
Semi-professionals	1.55 (1.04 - 2.31)	1.31 (0.85 - 2.03)
Routine non-manual workers	1.71 (1.24 - 2.37)	1.40 (0.96 - 2.06)
Manual workers	1.38 (1.00 - 1.90)	1.12 (0.76 - 1.66)
**Women**		
Managers or professionals	1.00	1.00
Semi-professionals	1.25 (1.02 - 1.54)	1.19 (0.92 - 1.53)
Routine non-manual workers	1.47 (1.24 - 1.76)	1.40 (1.11 - 1.76)
Manual workers	1.64 (1.28 - 2.11)	1.55 (1.16 - 2.08)
**Trajectory**	Low/decreasing vs. No OHS visits	
	Model 1*	Model 2**
**Men**		
Managers or professionals	1.00	1.00
Semi-professionals	1.32 (0.91 - 1.91)	1.45 (0.96 - 2.19)
Routine non-manual workers	1.31 (0.97 - 1.77)	1.53 (1.05 - 2.22)
Manual workers	1.26 (0.94 - 1.68)	1.50 (1.04 - 2.18)
**Women**		
Managers or professionals	1.00	1.00
Semi-professionals	1.48 (1.24 - 1.77)	1.71 (1.36 - 2.15)
Routine non-manual workers	1.33 (1.13 - 1.56)	1.67 (1.34 - 2.07)

For peer review only

Manual workers	1.30 (1.02 - 1.65)	1.65 (1.25 - 2.19)

**Trajectory**	High/recurrent vs. No OHS visits	
	Model 1*	Model 2**
**Men**		
Managers or professionals	1.00	1.00
Semi-professionals	3.21 (1.62 - 6.36)	2.60 (1.23 - 5.49)
Routine non-manual workers	3.99 (2.22 - 7.17)	2.92 (1.48 - 5.74)
Manual workers	5.02 (2.86 - 8.80)	3.56 (1.83 - 6.92)
**Women**		
Managers or professionals	1.00	1.00
Semi-professionals	2.13 (1.61 - 2.81)	2.29 (1.62 - 3.24)
Routine non-manual workers	2.53 (1.98 - 3.25)	2.28 (1.65 - 3.15)
Manual workers	3.10 (2.25 - 4.27)	2.71 (1.85 - 3.97)

Model 1* = Model adjusted for age and first language, Model 2** = M1 + education

Figure 1. Four occupational health service trajectories, based on registers covering the years 2004 to 2017 among 9762 the City of Helsinki employees aged 20-34 years. 1 = No visits, 2 = Low/increasing, 3 = Low/decreasing, 4 = High/recurrent use.

For peer review only

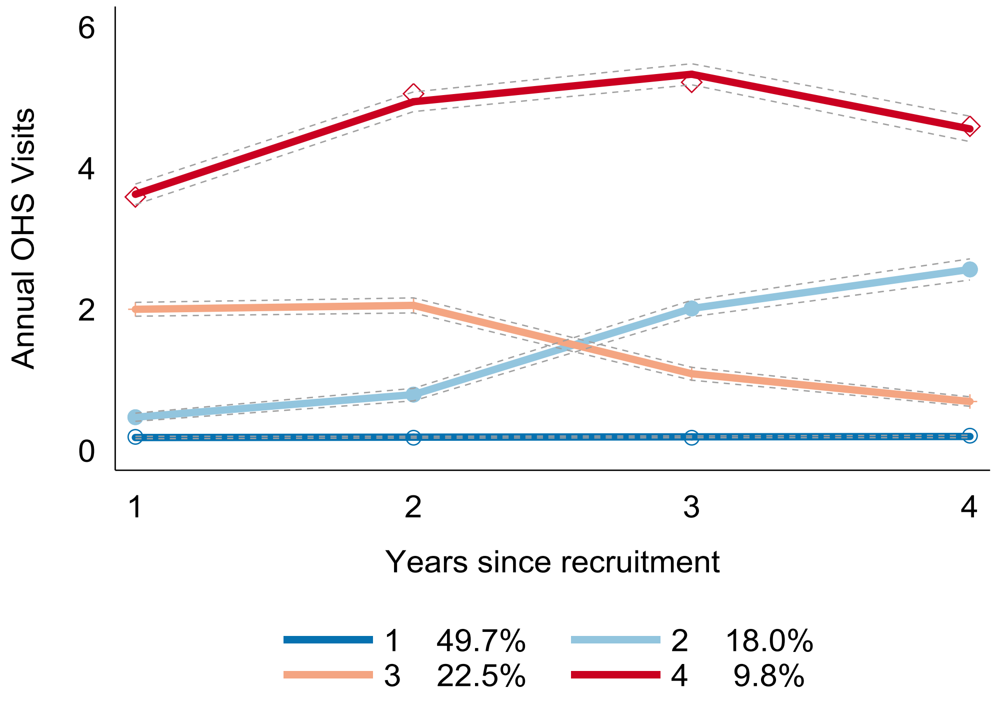

Figure 1. Four occupational health service trajectories, based on registers covering the years 2004 to 2017 among 9762 the City of Helsinki employees aged 20-34 years. 1 = No visits, 2 = Low/increasing, 3 = Low/decreasing, 4 = High/recurrent use.

For peer review only

Web-appendix 1. Comparison between those included* and those not included* in the present study, men

Men	Total		Included		Excluded	
	N	%	N	%	N	%
**Total**	6413	100.0	2454	100.0	3959	100.0
**Age**						
20 - 24	2170	33.8	618	25.2	1552	39.2
25 - 29	2507	39.1	1077	43.9	1430	36.1
30 - 34	1736	27.1	759	30.9	977	24.7
**Language**						
Finnish	5580	87.0	2154	87.8	3426	86.5
Swedish	274	4.3	81	3.3	193	4.9
Other	501	7.8	193	7.9	308	7.8
**Education**						
Basic education / Lower secondary	4604	71.8	1595	65.0	3009	76.0
Upper secondary	927	14.5	476	19.4	451	11.4
Higher education	882	13.8	383	15.6	499	12.6
**Occupational class**						
Managers or professionals	1331	20.8	594	24.2	737	18.6
Semi-professionals	587	9.2	286	11.7	301	7.6
Routine non-manual workers	1918	29.9	677	27.6	1241	31.3
Manual workers	2043	31.9	897	36.6	1146	28.9
**Working hours per week**						
32–45 h/wk	5003	78.0	1967	80.2	3036	76.7
<32 h/wk	1410	22.0	487	19.8	923	23.3
**Type of employment contract**						
Permanent contract	4603	71.8	2117	86.3	2486	62.8
Other contract type	1810	28.2	337	13.7	1473	37.2

*Included = employed by the City of Helsinki for at least 4 years and complete data on occupational position, Not included= employed by the City of Helsinki less than 4 years or incomplete data on occupational position.

For peer review only

Web-appendix 2. Comparison between those included* and those not included* in the present study, women

Women	Total		Included		Excluded	
	N	%	N	%	N	%
**Total**	16163	100.0	7308	100.0	8855	100.0
**Age**						
20 - 24	5609	34.7	2252	30.8	3357	37.9
25 - 29	6735	41.7	3152	43.1	3583	40.5
30 - 34	3819	23.6	1904	26.1	1915	21.6
**Language**						
Finnish	14028	86.8	6451	88.3	7577	85.6
Swedish	803	5.0	324	4.4	479	5.4
Other	1137	7.0	448	6.1	689	7.8
**Education**						
Basic education / Lower secondary	8785	54.4	3641	49.8	5144	58.1
Upper secondary	4420	27.3	2319	31.7	2101	23.7
Higher education	2958	18.3	1348	18.4	1610	18.2
**Occupational class**						
Managers or professionals	3674	22.7	1679	23.0	1995	22.5
Semi-professionals	2833	17.5	1538	21.0	1295	14.6
Routine non-manual workers	7561	46.8	3385	46.3	4176	47.2
Manual workers	1875	11.6	706	9.7	1169	13.2
**Working hours per week**						
32–45 h/wk	12678	78.4	5891	80.6	6787	76.6
<32 h/wk	3485	21.6	1417	19.4	2068	23.4
**Type of employment contract**						
Permanent contract	13059	80.8	6422	87.9	6637	75.0
Other contract type	3104	19.2	886	12.1	2218	25.0

*Included = employed by the City of Helsinki for at least 4 years and complete data on occupational position, Not included= employed by the City of Helsinki less than 4 years or incomplete data on occupational position.

For peer review only

STROBE Statement—checklist of items that should be included in reports of observational studies

Young Employees' Trajectories and Occupational Class Differences in Utilization of Primary Care Services Provided by Occupational Health Service

Hilla Sumanen, PhD
Jaakko Harkko, PhD
Kustaa Piha, PhD
Olli Pietiläinen, MSc
Ossi Rahkonen, Professor
Anne Kouvonen, Professor

	Item No	Recommendation	Done, page
**Title and abstract**	1	(*a*) Indicate the study's design with a commonly used term in the title or the abstract	3
		(*b*) Provide in the abstract an informative and balanced summary of what was done and what was found	3
**Introduction**			
Background/rationale	2	Explain the scientific background and rationale for the investigation being reported	4-5
Objectives	3	State specific objectives, including any prespecified hypotheses	5
**Methods**			
Study design	4	Present key elements of study design early in the paper	5
Setting	5	Describe the setting, locations, and relevant dates, including periods of recruitment, exposure, follow-up, and data collection	5-6
Participants	6	(*a*) *Cohort study*—Give the eligibility criteria, and the sources and methods of selection of participants. Describe methods of follow-up<br>*Case-control study*—Give the eligibility criteria, and the sources and methods of case ascertainment and control selection. Give the rationale for the choice of cases and controls<br>*Cross-sectional study*—Give the eligibility criteria, and the sources and methods of selection of participants	5-6
		(*b*) *Cohort study*—For matched studies, give matching criteria and number of exposed and unexposed<br>*Case-control study*—For matched studies, give matching criteria and the number of controls per case	
Variables	7	Clearly define all outcomes, exposures, predictors, potential confounders, and effect modifiers. Give diagnostic criteria, if applicable	6-7
Data sources/ measurement	8*	For each variable of interest, give sources of data and details of methods of assessment (measurement). Describe comparability of assessment methods if there is more than one group	6
Bias	9	Describe any efforts to address potential sources of bias	7, 14-

For peer review only - http://bmjopen.bmj.com/site/about/guidelines.xhtml

			15
Study size	10	Explain how the study size was arrived at	5-6
Quantitative variables	11	Explain how quantitative variables were handled in the analyses. If applicable, describe which groupings were chosen and why	7
Statistical methods	12	(*a*) Describe all statistical methods, including those used to control for confounding	7
		(*b*) Describe any methods used to examine subgroups and interactions	7
		(*c*) Explain how missing data were addressed	
		(*d*) *Cohort study*—If applicable, explain how loss to follow-up was addressed<br>*Case-control study*—If applicable, explain how matching of cases and controls was addressed<br>*Cross-sectional study*—If applicable, describe analytical methods taking account of sampling strategy	
		(*e*) Describe any sensitivity analyses	10

**Results**			**Done, page**
Participants	13*	(a) Report numbers of individuals at each stage of study—eg numbers potentially eligible, examined for eligibility, confirmed eligible, included in the study, completing follow-up, and analysed	5-8
		(b) Give reasons for non-participation at each stage	
		(c) Consider use of a flow diagram	
Descriptive data	14*	(a) Give characteristics of study participants (eg demographic, clinical, social) and information on exposures and potential confounders	7-8
		(b) Indicate number of participants with missing data for each variable of interest	
		(c) *Cohort study*—Summarise follow-up time (eg, average and total amount)	5-6, 8
Outcome data	15*	*Cohort study*—Report numbers of outcome events or summary measures over time	6, 8
		Case-control study—Report numbers in each exposure category, or summary measures of exposure	
		Cross-sectional study—Report numbers of outcome events or summary measures	
Main results	16	(*a*) Give unadjusted estimates and, if applicable, confounder-adjusted estimates and their precision (eg, 95% confidence interval). Make clear which confounders were adjusted for and why they were included	7-10
		(*b*) Report category boundaries when continuous variables were categorized	
		(*c*) If relevant, consider translating estimates of relative risk into absolute risk for a meaningful time period	
Other analyses	17	Report other analyses done—eg analyses of subgroups and interactions, and sensitivity analyses	10
**Discussion**			
Key results	18	Summarise key results with reference to study objectives	10-11
Limitations	19	Discuss limitations of the study, taking into account sources of potential bias or imprecision. Discuss both direction and magnitude of any potential bias	14-15
Interpretation	20	Give a cautious overall interpretation of results considering objectives, limitations, multiplicity of analyses, results from similar studies, and other relevant evidence	10-15
Generalisability	21	Discuss the generalisability (external validity) of the study results	10-15
**Other information**			

For peer review only

Funding	22	Give the source of funding and the role of the funders for the present study and, if applicable, for the original study on which the present article is based	16

*Give information separately for cases and controls in case-control studies and, if applicable, for exposed and unexposed groups in cohort and cross-sectional studies.

Note: An Explanation and Elaboration article discusses each checklist item and gives methodological background and published examples of transparent reporting. The STROBE checklist is best used in conjunction with this article (freely available on the Web sites of PLoS Medicine at http://www.plosmedicine.org/, Annals of Internal Medicine at http://www.annals.org/, and Epidemiology at http://www.epidem.com/). Information on the STROBE Initiative is available at www.strobe-statement.org.

For peer review only

For peer review only - http://bmjopen.bmj.com/site/about/guidelines.xhtml

BMJ Open

The association between socio-economic position and occupational health service utilization trajectories among young municipal employees in Finland

Journal:	*BMJ Open*
Manuscript ID	bmjopen-2018-028742.R2
Article Type:	Original research
Date Submitted by the Author:	24-Oct-2019
Complete List of Authors:	Sumanen, Hilla; South-Eastern Finland University of Applied Sciences, Health care and emergency care; University of Helsinki, Public Health<br>Harkko, Jaakko; University of Helsinki, Social Sciences<br>Piha, Kustaa ; University of Helsinki, Public Health<br>Pietilainen, Olli; University of Helsinki, Department of Public Health<br>Rahkonen, Ossi; University of Helsinki, Department of Public Health<br>Kouvonen, Anne; Helsingin Yliopisto, Faculty of Social Sciences; Queen's University Belfast,
**Primary Subject Heading**:	Health services research
Secondary Subject Heading:	Occupational and environmental medicine
Keywords:	health care visits, socioeconomic differences, young adults

SCHOLARONE™
Manuscripts

The association between socio-economic position and occupational health service utilization trajectories among young municipal employees in Finland

Hilla Sumanen, PhD 1,2,3

Jaakko Harkko, PhD 3

Kustaa Piha, MD, PhD 2

Olli Pietiläinen, MSc 2

Ossi Rahkonen, Professor 2

Anne Kouvonen, Professor 3, 4, 5

1. South-Eastern Finland University of Applied Sciences, Unit of Healthcare and Emergency care and Unit of Research, Development and Innovation
2. University of Helsinki, Faculty of Medicine, Department of Public Health
3. University of Helsinki, Faculty of Social Sciences
4. Research Institute of Psychology, SWPS University of Social Sciences and Humanities
5. Queen's University Belfast, Administrative Data Research Centre – Northern Ireland, Centre for Public Health

Correspondence to: Hilla Sumanen, Department of Public Health, University of Helsinki, Finland, P.O. Box 20 (Tukholmankatu 8B), FIN-00014, University of Helsinki, Finland; hilla.sumanen@helsinki.fi

For peer review only

What is already known about this subject?

This is the first study utilizing longitudinal occupational health service data. All the findings are novel.

What are the new findings?

We identified four occupational health service utilization groups among young employees: “No visits” (50%), “Low/increasing” (18%), “Low/decreasing” (22%) and ”High/recurrent” (10%) use. Lower occupational classes had a higher propensity for “High/recurrent” OHS utilization for both genders.

How might this impact on policy or clinical practice in the foreseeable future?

Preventive measures should be targeted particularly to the trajectory groups of “Low/increasing” and “High/recurrent” in order to intervene early. Occupational health service utilization should be more closely monitored among the two lowest occupational classes.

Running head: Young Employees’ Utilization of Occupational Health Service

For peer review only

Abstract

OBJECTIVES: To identify groups of municipal employees between the ages of 20 and 34 years with distinct utilization trajectories of primary care services provided by occupational health service (OHS), measured as the annual number of OHS visits, and to identify demographic and socio-economic risk factors that distinguish employees in the high utilization trajectory group(s).

METHODS: The present study is a retrospective register-based cohort study. All municipal employees of the City of Helsinki, Finland, aged 20-34 in the Helsinki Health Study (HHS), recruited from 2004 to 2013, with follow-up data for four years were included in the study (n = 9762). The outcome measure was group-based trajectories of OHS utilization, identified with a group-based trajectory analysis (GBTA). The demographic and socio-economic variables used to predict the outcome were age, first language, educational level and occupational class. The analyses were stratified by gender.

RESULTS: A large proportion of the young employees do not utilize OHS. Trajectory groups of “No visits” (50%), “Low/increasing” (18%), “Low/decreasing” (22%) and ”High/recurrent” (10%) use were identified. We found occupational class differences in OHS utilization patterns showing that lower occupational classes had a higher propensity for “High/recurrent” OHS utilization for both genders.

CONCLUSIONS: Preventive measures should be targeted particularly to the trajectory groups of “Low/increasing” and “High/recurrent” in order to intervene early. In addition, OHS utilization should be closely monitored among the two lowest occupational classes. More research with longitudinal OHS data is needed.

Key terms: Health care visits, Socioeconomic differences, Young adults, Occupational health

Strengths and limitations of this study

- This is the first study utilizing longitudinal occupational health service data
- The sample consisted all of 20-34-year-old employees of the City of Helsinki. The occupational health care policies are same for all these employees and have remained same during the study period of 2004-2017.
- Our study avoids a common limitation of previous occupational health service studies that are based on limited samples with data on only those attending the service or those responding to survey.
- Limitations of the data include the lack of diagnostic information and lifestyle factors.
- The lack of information about other primary care visits outside the occupational health service further limits the interpretation of the results.

For peer review only

Introduction

Finland has a unique occupational health service (OHS) system with statutory prevention of occupational health hazards (preventive services) and additionally purchased primary care services. OHS may be provided by employer's own OHS units, private clinics or public sector health centers with specifically trained occupational health physicians, nurses, physiotherapists and psychologists. In Finland, most employers purchase the additional primary care element for their employees. In 2017, 94% of the employees covered by statutory preventive OHS also had an access to primary care for any illness including all non-work related illnesses, paid by their employer and partly subsidized by the National Pension Fund [1]. OHSs are free for employees at the point of delivery and their accessibility is typically good, making them the main source and the preferred type of primary care for Finnish employees.

The utilization of OHS in Finland or elsewhere has been only scarcely studied, and especially studies using longitudinal data are lacking. There are few previous Finnish studies concentrating on the OHS primary care visits with cross-sectional study designs and from the viewpoint of service utilization over the course of 6 to 12 months. A recent study [2] with data from a large private Finnish OHS provider investigated the top 10% frequent attenders in primary care services in 2015. The results showed that frequent attendance was associated with female gender, being employed by a medium or a large company, working in the manufacturing industry, public administration or in health and social care services. In an earlier study with survey data (N=1636) from the Finnish working-age population [3], 57% of Finnish employees covered by the OHS primary care visited either their occupational health physician or nurse due to any illness during the 6-month period. In that study, those visits were strongly associated with chronic illness impacting occupational health and work ability. Both previous studies utilizing Finnish OHS data acknowledge the lack of research focusing to these unique services provided for the working population and identify the need for further study to identify service development needs and possibilities.

For peer review only

The current study utilizes longitudinal data from the own OHS unit of Finland's largest employer. The City of Helsinki offers same OHSs with primary care for all its employees (n=~38 000 per year) with no cost for the employees. The City of Helsinki employees have been the focus of the Helsinki Health Study [4] since 2000, but this is the first study using their OHS data. Our focus is on the younger employees and their OHS utilization, as previous studies have shown that they have a high prevalence of sickness absence (SA) [5] and there are already large socioeconomic differences in SA apparent in the younger age groups [6-7]. Reducing SA is high on employers' agenda. Service utilization information is important for planning preventive actions via targeted interventions or improving case management protocols [8-9] arranged by the OHS. According to extensive Finnish and international evidence, socioeconomic differences in SA are large [10-21], thus it would be important to monitor the differences in OHS utilization from the socioeconomic viewpoint and to be able to identify groups for interventions.

In our study, we aimed to identify developmental trajectories of OHS primary care service utilization among 20-34-year-old municipal employees of the City of Helsinki. In the second stage of the analysis we aimed to identify occupational class differences in belonging to different trajectory groups. We tested two hypothesis, first, that the distinct trajectories can be identified, and second, that occupational class gradient can be found in OHS utilization.

For peer review only

Methods

This is a retrospective register-based cohort study. The study is a part of the Helsinki Health Study on health and well-being among employees of the City of Helsinki, Finland [4]. The study included all Helsinki City employees aged 20-34 at the beginning of their first work contract with the City (N = 22,576) between 04/06/2004 and 04/19/2013. The selection of this age group was based on the Eurostat definition of young employees ,and on previous studies investigating the occurrence of

illness and sickness absence in employees of different ages [22, 23]. For each employee the follow-up started from their initial recruitment. Employees with incomplete data on occupational position (n= 754) and those with employment record for less than four years (n= 12,512) were excluded from the present study and we ended up with a sample size of n = 9,762. We excluded employees with less than four years of employment history, defined as being employed four years from the initial recruitment and at least 180 days of employment for each year after the recruitment, as we needed a long enough follow-up time to observe potential development trajectories. The descriptive characteristics of excluded subjects are found in web-appendixes 1 and 2. The length of the follow-up was measured as calendar days in employment.

The outcome of the study was OHS utilization trajectory. The OHS primary care services offered to the employees have remained same during the whole study period. The trajectory, i.e. the developmental course of OHS utilization, was measured from four consecutive data points indicating annual number of outpatient primary care visits for each employee. The number of visits ranged from 0 to 103. The demographic and socioeconomic variables used to predict the outcome were age, first language, educational level and occupational class. The analyses were stratified by gender.

We used four occupational class groups. Based on the socioeconomic classification of Statistics Finland and the occupational classification of the City of Helsinki [4,12], non-manual employees were divided into three groups based on skills requirements and supervisory status: Managers and professionals, semi-professionals, and routine non-manual employees. Managers have subordinates and they do managerial or administrative work, whereas professionals include employees with a university degree, such as physicians and teachers. Semi-professionals include occupations such as registered nurses and technicians. Routine non-manual employees include clerical employees and lesser-educated occupations particularly within the social and health care, such as child-minders and

For peer review only

care workers. The fourth occupational class, manual workers, include occupations for example from the fields of cleaning, kitchen work and public transport.

Age was measured at the beginning of the follow-up and was categorized into three groups: 20-24, 25-29 and 30-34-year-olds. First language was categorized as: Finnish, Swedish, Other. Education was classified into three levels: higher education (a master's or a doctoral degree), upper secondary (a Bachelor's degree), and lower secondary (upper-secondary school, vocational school) or basic education (comprehensive school).

The employer's personnel and occupational health care registers were used to obtain socio-demographic characteristics and information on OHS use. Educational level was obtained from annually updated Statistics Finland's registry of completed education and degrees, and was linked to the City of Helsinki personnel register.

Ethics

The study follows the Helsinki Health Study protocol in line with the University of Helsinki's guidelines and data legislation. The ethics committees of the Department of Public Health, the University of Helsinki and the health authorities of the City of Helsinki have approved the HHS study. The City of Helsinki has given permission for data linkage.

Patient and Public Involvement statement

No Patient or Public Involvement.

Statistical methods

Group-based trajectory modeling (GBTM) with Stata's *traj* command [24] was applied to identify clusters of individuals, or trajectory groups, with similar developmental trajectory on OHS utilization. The method assigns a subject to a trajectory group by assessing the probability of group

For peer review only

membership. The count variables were assumed to be Poisson-distributed and zero inflated Poisson models were applied. The ideal number of trajectory groups and trajectory shapes were assessed by four criteria suggested by the existing literature: Bayesian information criteria (BIC), posterior probabilities of trajectory group membership higher than .70, sizes of trajectory groups larger than 5% and a distinct interpretability of the identified trajectory groups [25,26]. Subsequently, multinomial logistic regression models using Stata's *mlogit* command were applied to investigate the role of occupational class as a predictor of the trajectory group membership. In a two-step-analysis, estimates are given for occupational class, first, adjusted for age and first language and, second, additionally adjusted for education. In the analyses, the trajectory-group membership is treated as the outcome, where the trajectory cluster indicating the lowest health care utilization was defined as the reference group and the other trajectories get the value 1 in each respective analyses. The results are given as relative risk ratios (RRRs) with their 95% confidence intervals (CIs). All statistical analyses were performed with Stata 15.

Results

Descriptive results

The study sample included 9762 the City of Helsinki employees aged between 20 and 34 at the beginning of the follow-up. 73%% of the employees were women. Among men, the yearly average of OHS visits was 1.03 (SD, 1.44) during the mean of 1341 days of follow-up. Among women, the yearly average was 1.16 (SD, 1.50) OHS visits in the mean of 1328 days. Of the subjects of the study, 2272 (23%) were managers or professionals, 1824 (19%) semi-professionals, 4064 (42%) routine non-manual workers, and 1602 (16%) manual workers (Tables 1-2). Managers/professionals had an equal gender distribution, semi-professionals and routine non-manual workers were more

For peer review only

often women, whereas men constituted the majority of manual workers. Occupational class was closely linked to educational attainment in both genders.

In trajectory analysis, a trajectory model consisting of four distinct trajectories including 1 trajectory with a linear, 1 with a quadratic, and 2 with a cubic shape showed the best fit using the BIC criterion (Figure 1). The largest identified trajectory group “No visits” (n = 5,106, 50%) represents those with less than 0.2 annual OHS visits over the four years of follow-up. There were two intermediate groups. The group labeled “Low/increasing” (n = 1,744, 18%) is characterized by low number of visits during the first two years followed by a slight increase in visits during the next two years. The group “Low/decreasing” (n = 2,238, 22%) follows a similar pattern as the group 2, but in a reverse order. The members of the both low groups averaged 1.5 annual OHS visits during the follow-up. The “High/recurrent” group (n = 976, 10%) consists of employees characterized by high levels of OHS visits, with an average of 4.6 visits per year, from the start of the employment to the end of the follow-up. The mean assignment probabilities were 0.93 for the "No visits", 0.81 for the "Low/increasing", 0.82 for the "Low/decreasing", and 0.92 for the "High/recurrent" trajectory groups, indicating a good model fit to the data. Of men, 54% belonged to the “No visits” trajectory and 8.4% to the “High/recurrent” trajectory group, whereas the corresponding figures for women were 50% and 10%, indicating a higher propensity for women to belong to the “High/recurrent” trajectory group. The assignment of the members of different occupational classes to different OHS trajectories followed the socio-economic gradient. Of managers or professionals, 59% belonged to the “No visits” trajectory and 5% to the “High/recurrent” trajectory. The corresponding figures for semi-professionals were 48% and 10%, for routine non-manual workers 48% and 11%, and for manual workers 49% and 13%, respectively.

*** Figure 1 ***

For peer review only

*** Table 1 ***

*** Table 2 ***

OHS utilization trajectories by occupational class

Occupational class was a strong independent predictor for the OHS utilization trajectories, as demonstrated in Table 3. The likelihood of belonging to the "High/recurrent" trajectory was increased for those being in a lower occupational classes when compared to the employees in managerial or professional positions. For both women and men, the risk for belonging to the "High/recurrent" trajectory was highest for manual workers, followed by routine non-manual workers and semi-professionals.

The independent effect of occupational class remained after adjustment for all covariates including age, first language and education. The association was most evident in the "High/recurrent" trajectory. The relative risk for this group membership was 2.92 (95% CI, 1.48 – 5.74) for male routine non-manual workers and 3.56 (95% CI, 1.83 – 6.92) for male manual workers. The corresponding figures for women were 2.28 (95% CI, 1.65 – 3.15) and 2.71 (95% CI, 1.85 – 3.97), respectively. The results indicate that a proportion of the association between occupational class and belonging to the "High/recurrent" trajectory is dependent on the lower educational attainment of the members of the lower occupational classes. The results comparing the two low trajectories with the "No visits" OHS trajectory were less clear in terms of statistical significance. Whereas all estimates but one in "Low/decreasing" vs "No visits" comparison remained statistically significant after full adjustment, four out of "Low/increasing" vs "No visits" comparisons become statistically non-significant in the final model. The observed excess risks generated by occupational class were thus smaller in both "Low" trajectories compared to "High/recurrent" trajectory. Notably, the relative

For peer review only

risks related to the membership of these middle trajectories were not manifested in a dose-exposure manner as was the case with the "High/recurrent" trajectory.

*** Table 3 ***

Sensitivity analyses

To assess the extent the parameter estimates were sensitive to potential errors in model specification and data, four types of sensitivity analyses were performed. First, we reproduced estimates from the original data with bootstrap resampling (1000 replications). Second, we reproduced the results with logistic regression analyses defining the high-utilization group as those whose total number of OHS visits during the follow-up period was 10 or more (n=1,484) was compared with those with no or just one visit (n= 3,993). Third, we ran the analysis with reversed class order in order to identify whether semi-professionals were less likely to be in the high trajectory group compared to routine workers (web-appendix 3). These sensitivity analyses indicated robustness of our inference about the relationship between occupational class and OHS utilization trajectories. Fourth, we performed a sensitivity analysis where variables on part-time work and fixed-term contract were included, but they changed the estimates only modestly (data not shown).

Discussion

In this study we identified developmental trajectories and socioeconomic differences in OHS primary care service utilization among 20 to 34-year-old employees of the City of Helsinki from 2004 to 2017. Our key results were: 1) Half of the young employees did not use OHS to any considerable extent; 2) Higher occupational classes utilized less OHS; 3) Four trajectory groups,

For peer review only - http://bmjopen.bmj.com/site/about/guidelines.xhtml

that is, “No visits”, ”Low/increasing”, “Low/decreasing” and “High/recurrent”, were identified. 4) The trajectory group of “High/recurrent” included a larger number of lower class workers especially among men, and the differences were large also among women. 5) Occupational class differences in “Low/decreasing” group were evident in both genders. 6) Only in women there were some occupational class differences in belonging to the trajectory group of “Low-increasing OHS utilization”.

Our results highlight the significance of socioeconomic gradient in OHS utilization that was visible both in men and women. The percentage of those who had no visits was the highest among managers and professionals and the proportion of no visits decreased when going down the occupational class ladder. Respectively, high and recurrent use was smallest among managers and professionals, and increased with decreasing occupational class, this type of use being the most common among manual workers. A larger proportion of men had no visits at all in each occupational class, thus the women used the health services more, in line with earlier findings [2]. In a similar way, the high and recurrent use was higher among women than among men. In the present context, primary care visits can be interpreted as an indicator of incidence of acute illnesses, as the Finnish OHS system distinguish visits related to occupational health hazards. The present results concerning primary care visits are in line with previous findings from our own and other studies showing the socioeconomic differences in sickness absence among employees and the gender differences in sickness absence, i.e. women having more absence than men [7,10,14,18,21]. It can be assumed that large number of OHS visits precede sickness absence. [27].

Stratified analyses indicated gender differences in OHS utilization. According to our results, among men the occupational class differences disappeared after full adjustment in the trajectory group of “Low/increasing”. This implies that the initial differences are associated with the type of work tasks. In contrast, after full adjustment, among women the employees in the two lowest occupational classes had a higher risk for belonging to this trajectory group. In the trajectory group

For peer review only

of "Low/decreasing" the differences were initially similar among the three lowest occupational classes. However, the differences modestly increased after full adjustment, implying that there are several factors associated with the low OHS utilization. This was seen among both genders.

The trajectory group of "High/recurrent" is perhaps the most interesting group alongside with the "Low/increasing" group in terms of costs and possible preventive opportunities. According to our results the occupational class differences in this group are steep especially among men, and also large among women. After full adjustment the differences decreased more in men, suggesting that the initial differences are more associated with work tasks among them. However, the differences remained high in both genders after adjustments. Studies regarding socioeconomic differences in sickness absence have also found that the differences are steeper among men [7,17,18], but the former studies mostly concentrate on older employees.

The differences in physical and psychosocial demands between occupational classes are important to take into account when interpreting the results. Manual workers have more physically demanding jobs, which may affect their need for primary care services. Adverse working conditions may cause ill-health and need of health services as milder even health difficulties may prevent these employees from working. Employees in higher occupational classes typically have more complex and mentally demanding jobs [28]. In studies examining the socioeconomic differences in sickness absence, physical working conditions have been found to be the strongest explanatory factor [12,16]. However, employees in our study are fairly young and thus adverse physical or psychosocial working conditions might not have yet affected their health, as health-related effects usually increase with age. In addition, the unique OHS system where the visits associated with occupational health hazards (preventive services) are recorded separately from primary care visits may contribute to the differences seen in our results. For example visits with more chronic work-related reason are usually not recorded as primary care visits. Thus, the overall utilization of OHS requires further research.

For peer review only

Our study indicates that service use patterns might recognize vulnerable groups more precisely than just belonging to certain occupation or occupational class may do. Despite this, the two lowest occupational classes may need extra attention based on their OHS utilization patterns. Case management protocols are essential in coordinating patient-centered care path which also saves costs [8,9,29]. Among younger employees, timely treatment is highly important, as it might prevent the worsening of their condition. OHS should identify those employees who use services a lot.

Previous longitudinal studies using OHS data are lacking, but recent studies have showed that frequent utilization of OHS was associated with psychiatric problems and musculoskeletal disorders [2], whereas the latter also predicts persistent frequent utilization [30]. Furthermore, frequent utilization has increased the risk of long sickness absence [31] and disability pension [32]. These associations highlight the need of identifying those in risk for more severe illness and work disability at an early stage, and information on the different utilization trajectories with identified occupational class differences supports these preventive actions. Moreover, some comparisons can be made with the studies investigating frequent attenders in primary care. Frequent attenders in primary care in the general population have been studied particularly in Netherlands and in Sweden using questionnaire surveys and record linkage. These studies had participants from a wide range of sociodemographic backgrounds and they consider only visits to general practitioners, thus their direct comparability to OHS utilization is difficult. In addition, the definition of frequent attender varies between studies [33]. However, previous studies have found out that frequent attenders have multiple reasons for presenting [34], but overall chronic illnesses [33], somatic diseases and symptoms [35,36] and especially psychiatric problems [35] have been associated with more frequent primary care service use. Frequent attenders have more health discomfort, low mastery, and they may be more vulnerable for stressful life events due to inadequate coping strategies [27,37,38]. In line with the study by Reho et al. [31,32], frequent attenders are a high-risk group for long-term sickness absence and disability pension [27]. According to two previous Dutch studies

For peer review only

one out of every seven 1-year frequent attenders becomes persistent frequent attender and six out of seven are transient frequent attenders [35,39]. Based on this previous evidence the inclusion of diagnostic information would be important in future studies of OHS utilization. However, from the methodological viewpoint both the Dutch and Swedish research groups point out that age should be taken into account when studying the frequent attenders, as not only the reasons for high service utilization but also what constitutes high use highly differ by age group [36,40].

Methodological considerations

The registers used in this study are reliable and comprehensive. We focused on all occupational groups within the largest employer in Finland and the sample consisted all of 20-34-year-old employees within this organization. The occupational health care policies are same for all these employees and have remained same during the study period of 2004-2017. Our study avoids a common limitation of OHS studies that are based on limited samples with data on only those attending the service (e.g. [2]) or those responding to survey [3]. Another advantage of this study is that we could make inferences based on longitudinal cohort data instead of relying on cross-sectional evidence. Limitations of the data include the lack of information of diagnoses, physical and psychosocial working conditions, lifestyle factors and any primary care visits outside the OHS. Unfortunately, our data does not extend beyond the employees' current employment contracts. In addition, people with initial poor health may attain lower educational level and end up in lower occupational positions. Moreover, excluding those with employment record for less than four years (n= 12,512) due to the need of long enough follow-up time reduced particularly the number of youngest employees (web-appendixes 1 and 2). A further limitation is that the initial occupational classes might have changed during the follow-up for due to promotion or other changes in the employment.

The OHS system in Finland is unique, thus comparison to other countries is difficult. The principle of primary care use is similar as in the general practice (GP) or family doctor setting in most other

For peer review only

Western countries, but the patient population differs to some extent from ours in terms of demographics and employment status. In Finland, the employer offers (most employers do) those services and thus OHS is the main source for primary care for employees due to being free at the point of delivery and enabling an easy access. However, even within Finland, different employers can have different policies in terms of provision of primary care services. Nevertheless, our results can be broadly generalized to Finnish public sector employees.

The present study is to our knowledge the first one that used longitudinal latent class analysis aiming to capture OHS utilization as a complex longitudinal phenomenon. GBTM approach mixes the application of formal statistical criteria and subjective evaluation in model fitting [24]. One of its strength is that it allowed us to identify high OHS utilization over time. It is a limitation that those who left the City of Helsinki within the first four years of their employment were lost to follow-up. Another benefit is that GBTM is capable of identifying different OHS trajectories within subjects that appear similar in terms of summary statistics. In this study we were able to distinguish between two "low" trajectories, which may allow for better planning of targeted prevention measures. However, we want to highlight that by this methodology it cannot be ascertained that the observed subgroups are distinct population subgroups. As in case of any latent trajectory class analyses, there is a possibility that the data could be interpreted as homogenous but non-normal [41]. We find, however, the obtained groups to be realistic and the results applicable in terms of real-life interpretations. However, further analysis with longer follow-up would be important to confirm the trajectories found.

Conclusions

We used GBTM for distinguishing four different developmental trajectories in OHS primary care service utilization among 20-34-year-old employees of the City of Helsinki. We found that

For peer review only

occupational class differences exist in the utilization development trajectories. A large proportion of the young employees do not use OHS primary care services and non-use is the most common among the highest occupational class. Especially trajectories where the utilization has grown or been high throughout the follow-up had large occupational class differences, which followed the socioeconomic gradient. Identifying high utilization patterns is important as ten per cent of employees that may be labeled high and recurrent users account for 40% of the all OHS consultations.

Our results show several important points for policy makers, as well as occupational and primary health care personnel, not only in Finland but also in countries with different primary care and occupational health care systems. According to our results preventive measures should be considered particularly among the trajectory groups of "Low/increasing" and "High/recurrent" health care utilization. In addition, special attention should be paid to the lowest occupational classes, and their OHS utilization should be closely monitored by the occupational health care/primary care in order to identify those in need for extra support. Case management protocols should be further developed and resources targeted in order to develop and maintain the health care system where early support is been given to those identified being in risk for subsequent work disability. As the preventive measures are done in practice, research should follow their success and produce evidence based development suggestions. In addition, OHS and primary care utilization requires more longitudinal research in order to target resources and preventive measures.

For peer review only

Author contributions

HS, AK & JH designed the study. HS mainly wrote the manuscript with contributions and comments from all the other authors (JH, KP, OP, OR & AK), JH did the statistical analyses and wrote the results section and OP commented those. All the authors have approved the final version of the manuscript.

Conflicts of Interest

Authors declare no conflicts of interest.

Funding

This study was funded by the Finnish Work Environment Fund (grant 117321) and the Academy of Finland (grant 315343). AK was funded by the Economic and Social Research Council (ESRC) (grant ES/L007509/1 and ES/S00744X/1). OR was funded by the Academy of Finland (grant 1294514). The funders had no involvement to the preparation of the manuscript.

Data sharing statement

Data cannot be shared publicly because it contains confidential medical information and the study participants have not given their permission to data sharing. Data are kept at the University of Helsinki computers and are available upon agreement with the Helsinki Health Study for researchers who meet the criteria for access to confidential data.

For peer review only

References

1. Kelasto-reports. 2019. Kelan työterveyshuoltotilasto 2017 (in Finnish). Official statistics of Finland. https://helda.helsinki.fi/bitstream/handle/10138/303534/Kelan_tyoterveyshuoltotilasto_2017.pdf?sequence=1&isAllowed=y. (Accessed 6 Aug 2019).

2. Reho T, Atkins S, Talola N, Viljamaa M, Sumanen M, Uitti J. Frequent attenders in occupational health primary care: A cross-sectional study. Scand J Public Health 2018;1:1403494818777436.

3. Kimanen A, Rautio M, Manninen P, Räsänen K, Husman P, Husman K. Primary care visits to occupational health physicians and nurses in Finland. Scand J Public Health 2011;39:525-32.

4. Lahelma E, Aittomäki A, Laaksonen M, Lallukka T, Martikainen P, Piha K, et al. Cohort Profile: The Helsinki Health Study. Int J Epidemiol 2013;42:722–30.

5. Sumanen H, Pietiläinen O, Lahti J, Lahelma E, Rahkonen O. Sickness absence among young employees: trends from 2002 to 2013. J Occup Health 2015;57:474-81.

6. Sumanen H, Lahelma E, Lahti J, Pietiläinen O, Rahkonen O. Educational differences in sickness absence trends among young employees from 2002 to 2013 in Helsinki, Finland. BMJ Open 2016; 6:e008550.

7. Sumanen H, Lahelma E, Pietiläinen O, Rahkonen O. The Magnitude of Occupational Class Differences in Sickness Absence: 15-Year Trends among Young and Middle-Aged Municipal Employees. Int J Environ Res Public Health 2017;14:E625.

8. AAOHN. Case Management: The Occupational and Environmental Health Nurse Role. Workplace Health Saf 2013;61:8.

9. Robinson M, Fisher TF, Broussard K. Role of Occupational Therapy in Case Management and Care Coordination for Clients With Complex Conditions. Am J Occup Ther 2016;70:7002090010p1-7002090010p6.

For peer review only

10. Piha K, Laaksonen M, Martikainen P, Rahkonen O, Lahelma E. Interrelationships between education, occupational class, income and sickness absence. Eur J Public Health 2010;20:276-80.

11. Piha K, Martikainen P, Rahkonen O, Roos E, Lahelma E. Trends in socioeconomic differences in sickness absence among Finnish municipal employees 1990-99. Scand J Public Health 2007;35:348-55.

12. Laaksonen M, Piha K, Rahkonen O, Martikainen P, Lahelma E. Explaining occupational class differences in sickness absence: Results from middle-aged municipal employees. J Epidemiol Community Health 2010;64:802–7.

13. Sumanen H, Pietiläinen O, Lahti J, Lahelma E, Rahkonen O. Interrelationships between education, occupational class and income as determinants of sickness absence among young employees in 2002-2007 and 2008-2013. BMC Public Health 2015;15:332.

14. Pekkala J, Blomgren J, Pietiläinen O, Lahelma E, Rahkonen O. Occupational class differences in diagnostic-spesific sickness absence: A register-based study in the Finnish population. BMC Public Health 2017;17:670.

15. Feeney A, North F, Head J, Canner R, Marmot M. Socioeconomic and sex differentials in reason for sickness absence from the Whitehall II study. Occup Environ Med 1998;55:91-8.

16. Christensen K, Labriola M, Lund T, Kivimäki M. Explaining the social gradient in long-term sickness absence: A prospective study of Danish employees. J Epidem Community Health 2008;62:181–3.

17. Hansen H, Ingebrigtsen T. Social class and sickness absence in Norway. Acta Sociologica 2008;51:309–27.

18. Löve J, Hensing G, Holmgren K, Toren K. Explaining the social gradient in sickness absence: A study of a general working population in Sweden. BMC Public Health 2013;13:545-2458-13-545.

For peer review only

19. Vahtera J, Virtanen P, Kivimäki M, Pentti J. Workplace as an origin of health inequalities. J Epidemiol Community Health 1999;53:399-407.

20. Morikawa Y, Martikainen P, Head J, Marmot M, Ishizaki M, Nakagawa H. A comparison of socio-economic differences in long-term sickness absence in a Japanese cohort and a British cohort of employed men. Eur J Public Health 2004;14:413-6.

21. Kaikkonen R, Härkänen T, Rahkonen O, Gould R, Koskinen S. Explaining educational differences in sickness absence: a population-based follow-up study. Scand J Work Environ Health 2015;41:338-46.

22. Sumanen H. Work disability among young employees: Changes over time and socioeconomic differences. Dissertationes Scholae Doctoralis Ad Sanitatem Investigandam Universitatis Helsinkiensis; nro 18/2016.

23. Eurostat. 2019. Participation of Young People in Education and the Labour Market. https://ec.europa.eu/eurostat/statistics-explained/index.php?title=Participation_of_young_people_in_education_and_the_labour_market&oldid=433402#Participation_of_young_persons_in_formal_education_and_in_the_labour_market. (Accessed 6 Aug 2019)

24. Jones BL, Nagin DS. 2012. A Stata Plugin for Estimating Group-Based Trajectory Models. https://ssrc.indiana.edu/doc/wimdocs/2013-03-29_nagin_trajectory_stata-plugin-info.pdf (accessed 1 Jun 2018)

25. Nagin D. 2005. Group-Based Modeling of Development. Cambridge, Mass: Harvard University Press.

26. Nagin DS, Odgers CL. Group-Based Trajectory Modeling in Clinical Research. Annu Rev Clin Psychol 2010;6:109–38.

For peer review only

27. Bergh H, Baigi A, Månsson J, Mattsson B, Marklund B. Predictive factors for long-term sick leave and disability pension among frequent and normal attenders in primary health care over 5 years. Public Health 2007;121:25-33.

28. Nieuwenhuijsen K, Verbeek JH, de Boer AG, Blonk RW, van Dijk FJ: Predicting the duration of sickness absence for patients with common mental disorders in occupational health care. Scand J Work Environ Health 2006;32:67-74.

29. Golden R. The importance of collaboration. CMSA Today 2015;4:21-2.

30. Reho T, Atkins S, Talola N, Sumanen M, Viljamaa M, Uitti J. Comparing occasional and persistent frequent attenders in occupational health primary care - a longitudinal study. BMC Public Health 2018;26:1291.

31. Reho TTM, Atkins SA, Talola N, Sumanen MPT, Viljamaa M, Uitti J. Occasional and persistent frequent attenders and sickness absences in occupational health primary care: a longitudinal study in Finland. BMJ Open 2019;19:e024980.

32. Reho TTM, Atkins SA, Talola N, Sumanen MPT, Viljamaa M, Uitti J. Frequent attenders at risk of disability pension: a longitudinal study combining routine and register data. Scand J Public Health 2019;11:1403494819838663.

33. Vedsted P, Christensen MB. Frequent attenders in general practice care: a literature review with special reference to methodological considerations. Public Health 2005;119:118-37.

34. Smits FT, Brouwer HJ, Schene AH, van Weert HC, Ter Riet G. Is frequent attendance of longer duration related to less transient episodes of care? A retrospective analysis of transient and chronic episodes of care. BMJ Open 2016;6:e012563.

For peer review only

35. Smits FT, Brouwer HJ, ter Riet G, van Weert HC. Epidemiology of frequent attenders: a 3-year historic cohort study comparing attendance, morbidity and prescriptions of one-year and persistent frequent attenders. BMC Public Health 2009:9;36.

36. Bergh H, Marklund B. Characteristics of frequent attenders in different age and sex groups in primary health care. Scand J Prim Health Care 2003;21:171-7.

37. Bergh H, Baigi A, Fridlund B, Marklund B. Life events, social support and sense of coherence among frequent attenders in primary health care. Public Health 2006;120:229-36.

38. Smits FT, Brouwer HJ, Zwinderman AH, Mohrs J, Schene AH, van Weert HC, ter Riet G. Why do they keep coming back? Psychosocial etiology of persistence of frequent attendance in primary care: a prospective cohort study. J Psychosom Res 2014;77:492-503.

39. Smits FT, Brouwer HJ, van Weert HC, Schene AH, ter Riet G. Predictability of persistent frequent attendance: a historic 3-year cohort study. Br J Gen Pract 2009;59:e44-50.

40. Smits FT, Mohrs JJ, Beem EE, Bindels PJ, van Weert HC. Defining frequent attendance in general practice. BMC Fam Pract.2008;15:21.

41. Bauer DJ, Curran PJ. Distributional Assumptions of Growth Mixture Models: Implications for Overextraction of Latent Trajectory Classes. Psychological Methods 2003;8:338-63.

For peer review only

Table 1. Descriptive statistics of the four occupational classes among 2,454 male The City of Helsinki employees aged 20-34 years. Results are based on register data covering the years from 2004 to 2017.

	Total				Occupational class							
			OHS visits p.a.		*Managers or professionals*		*Semi-professionals*		*Routine non-manual workers*		*Manual workers*	
	N	% / (sd.)	N	1 / 1 000	*n*	*% / (sd.)*	*n*	*% / (sd.)*	*n*	*% / (sd.)*	*n*	*% / (sd.)*
**Total**	2454	100.0	2528	1030.3	594	100.0	286	100.0	677	100.0	897	100.0
The length of the follow-up in days, average (sd.)	1341	(252)			1371	(204)	1382	(191)	1290	(292)	1348	(260)
OHS visits per annum, 1 / 1000 (sd.)	1030.3	(1435.3)			705.4	(939.4)	996.5	(1151.3)	1121.5	(1482.1)	1187.3	(1695.8)
**Outcome: Trajectory group**												
1. No OHS visits	1337	54.5	225	168.5	374	63.0	148	51.7	349	51.6	466	52.0
2. Low/increasing	420	17.1	626	1489.3	91	15.3	54	18.9	131	19.4	144	16.1
3. Low/decreasing	496	20.2	728	1468.2	113	19.0	63	22.0	138	20.4	182	20.3
4. High/recurrent	201	8.2	949	4722.6	16	2.7	21	7.3	59	8.7	105	11.7
Covariates												
**Age (years)**												
20 - 24	618	25.2	684	1107.2	23	3.9	38	13.3	221	32.6	336	37.5
25 - 29	1077	43.9	996	924.6	299	50.3	152	53.1	296	43.7	330	36.8
30 - 34	759	30.9	848	1117.6	272	45.8	96	33.6	160	23.6	231	25.8
**First language**												
Finnish	2154	87.8	2219	1029.9	499	84.0	272	95.1	589	87.0	794	88.5

For peer review only

Swedish	81	3.3	36	438.3	47	7.9	2	0.7	24	3.5	8	0.9
Other	193	7.9	256	1327.7	24	4.0	12	4.2	62	9.2	95	10.6
**Education**												
Basic education / Lower secondary	1595	65.0	1807	1132.8	136	22.9	115	40.2	530	78.3	814	90.7
Upper secondary	476	19.4	453	951.2	126	21.2	149	52.1	130	19.2	71	7.9
Higher education	383	15.6	269	701.7	332	55.9	22	7.7	17	2.5	12	1.3

For peer review only

Table 2. Descriptive statistics of the four occupational classes among 7,308 female The City of Helsinki employees aged 20-34 years. Results are based on register data covering the years from 2004 to 2017.

	**Total**				**Occupational class**							
			**OHS visits p.a.**		***Managers or professionals***		***Semi-professionals***		***Routine non-manual workers***		***Manual workers***	
	N	% / (sd.)	N	1 / 1 000	*n*	*% / (sd.)*	*n*	*% / (sd.)*	*n*	*% / (sd.)*	*n*	*% / (sd.)*
**Total**	7308	100.0	8470	1158.9	1678	100.0	1538	100.0	3387	100.0	705	100.0
The length of the follow-up in days, average (sd.)	1328	(254)			1326	(244)	1375	(202)	1310	(272)	1322	(281)
OHS visits per annum, 1 / 1000 (sd.)	1158.9	(1499.2)			850.9	(1114.0)	1141.7	(1357.4)	1268.7	(1627.4)	1402.5	(1808.0)
**Outcome: Trajectory group**												
1. No OHS visits	3623	49.6	644	177.7	972	57.9	734	47.7	1599	47.2	318	45.1
2. Low/increasing	1281	17.5	1922	1500.2	264	15.7	249	16.2	627	18.5	141	20.0
3. Low/decreasing	1651	22.6	2455	1487.0	347	20.7	397	25.8	759	22.4	148	21.0

4. High/recurrent	753	10.3	3449	4580.3	95	5.7	158	10.3	402	11.9	98	13.9
Covariates												
**Age**												
20 - 24	2252	30.8	2693	1195.6	142	8.5	443	28.8	1412	41.7	255	36.2
25 - 29	3152	43.1	3565	1130.9	959	57.2	693	45.1	1233	36.4	267	37.9
30 - 34	1904	26.1	2212	1161.9	577	34.4	402	26.1	742	21.9	183	26.0
**Language**												
Finnish	6451	88.3	7627	1182.3	1403	83.6	1422	92.5	3020	89.2	606	86.0
Swedish	324	4.4	307	947.5	158	9.4	51	3.3	109	3.2	6	0.9
Other	448	6.1	479	1068.6	40	2.4	64	4.2	252	7.4	92	13.0
**Education**												
Basic education / Lower secondary	3641	49.8	4667	1281.8	273	16.3	232	15.1	2541	75.0	595	84.4
Upper secondary	2319	31.7	2565	1105.9	270	16.1	1211	78.7	755	22.3	83	11.8
Higher education	1348	18.4	1238	918.4	1135	67.6	95	6.2	91	2.7	27	3.8

For peer review only

Table 3. Multinomial logistic regression on occupational class as a determinant of occupational health service (OHS) trajectories among 9762 City of Helsinki employees aged 20-34 years. Results are based on register data covering the years from 2004 to 2017.

	OHS trajectory comparison	
**Trajectory**	Low/increasing vs. No OHS visits	
	Model 1*	Model 2**
**Men**		
Managers or professionals	1.00	1.00
Semi-professionals	1.55 (1.04 - 2.31)	1.31 (0.85 - 2.03)
Routine non-manual workers	1.71 (1.24 - 2.37)	1.40 (0.96 - 2.06)
Manual workers	1.38 (1.00 - 1.90)	1.12 (0.76 - 1.66)
**Women**		
Managers or professionals	1.00	1.00
Semi-professionals	1.25 (1.02 - 1.54)	1.19 (0.92 - 1.53)
Routine non-manual workers	1.47 (1.24 - 1.76)	1.40 (1.11 - 1.76)
Manual workers	1.64 (1.28 - 2.11)	1.55 (1.16 - 2.08)

**Trajectory**	Low/decreasing vs. No OHS visits	
	Model 1*	Model 2**
**Men**		
Managers or professionals	1.00	1.00
Semi-professionals	1.32 (0.91 - 1.91)	1.45 (0.96 - 2.19)
Routine non-manual workers	1.31 (0.97 - 1.77)	1.53 (1.05 - 2.22)
Manual workers	1.26 (0.94 - 1.68)	1.50 (1.04 - 2.18)
**Women**		
Managers or professionals	1.00	1.00
Semi-professionals	1.48 (1.24 - 1.77)	1.71 (1.36 - 2.15)
Routine non-manual workers	1.33 (1.13 - 1.56)	1.67 (1.34 - 2.07)

For peer review only

Trajectory	High/recurrent vs. No OHS visits	
Manual workers	1.30 (1.02 - 1.65)	1.65 (1.25 - 2.19)

Trajectory	High/recurrent vs. No OHS visits	
	Model 1*	Model 2**
**Men**		
Managers or professionals	1.00	1.00
Semi-professionals	3.21 (1.62 - 6.36)	2.60 (1.23 - 5.49)
Routine non-manual workers	3.99 (2.22 - 7.17)	2.92 (1.48 - 5.74)
Manual workers	5.02 (2.86 - 8.80)	3.56 (1.83 - 6.92)
**Women**		
Managers or professionals	1.00	1.00
Semi-professionals	2.13 (1.61 - 2.81)	2.29 (1.62 - 3.24)
Routine non-manual workers	2.53 (1.98 - 3.25)	2.28 (1.65 - 3.15)
Manual workers	3.10 (2.25 - 4.27)	2.71 (1.85 - 3.97)

Model 1* = Model adjusted for age and first language, Model 2** = M1 + education

Figure 1. Four occupational health service trajectories, based on registers covering the years 2004 to 2017 among 9762 the City of Helsinki employees aged 20-34 years. 1 = No visits, 2 = Low/increasing, 3 = Low/decreasing, 4 = High/recurrent use.

For peer review only

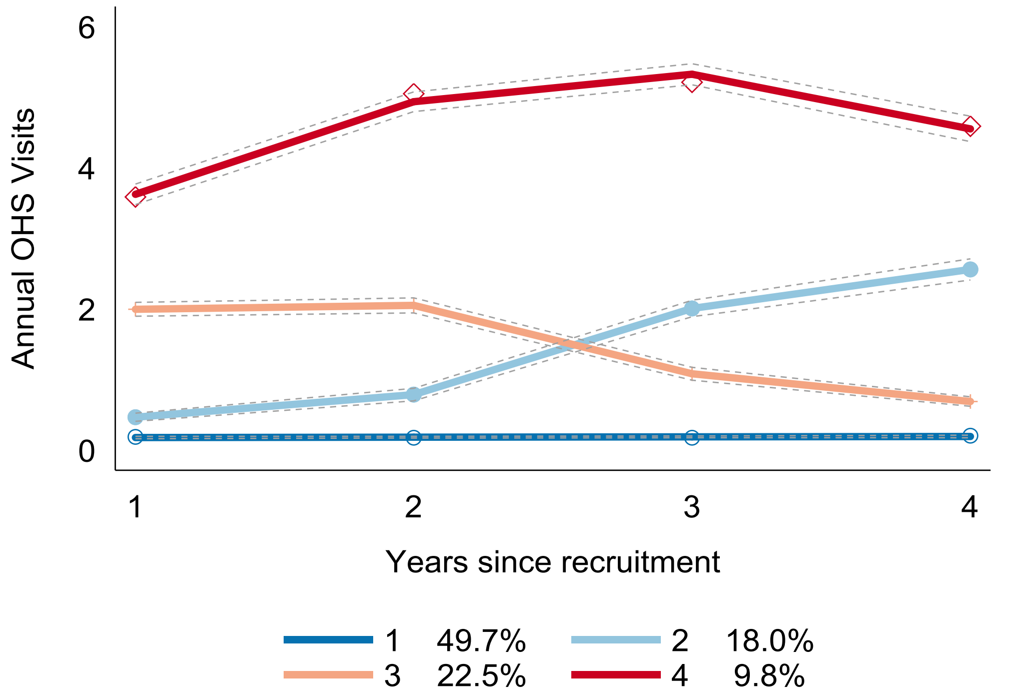

Figure 1. Four occupational health service trajectories, based on registers covering the years 2004 to 2017 among 9762 the City of Helsinki employees aged 20-34 years. 1 = No visits, 2 = Low/increasing, 3 = Low/decreasing, 4 = High/recurrent use.

Web-appendix 1. Comparison between those included* and those not included* in the present study, men

**Men**	**Total**		**Included**		**Excluded**	
	N	%	N	%	N	%
**Total**	6413	100.0	2454	100.0	3959	100.0
**Age**						
20 - 24	2170	33.8	618	25.2	1552	39.2
25 - 29	2507	39.1	1077	43.9	1430	36.1
30 - 34	1736	27.1	759	30.9	977	24.7
**Language**						
Finnish	5580	87.0	2154	87.8	3426	86.5
Swedish	274	4.3	81	3.3	193	4.9
Other	501	7.8	193	7.9	308	7.8
**Education**						
Basic education / Lower secondary	4604	71.8	1595	65.0	3009	76.0
Upper secondary	927	14.5	476	19.4	451	11.4
Higher education	882	13.8	383	15.6	499	12.6
**Occupational class**						
Managers or professionals	1331	20.8	594	24.2	737	18.6
Semi-professionals	587	9.2	286	11.7	301	7.6
Routine non-manual workers	1918	29.9	677	27.6	1241	31.3
Manual workers	2043	31.9	897	36.6	1146	28.9
**Working hours per week**						
32–45 h/wk	5003	78.0	1967	80.2	3036	76.7
<32 h/wk	1410	22.0	487	19.8	923	23.3
**Type of employment contract**						
Permanent contract	4603	71.8	2117	86.3	2486	62.8
Other contract type	1810	28.2	337	13.7	1473	37.2

*Included = employed by the City of Helsinki for at least 4 years and complete data on occupational position, Not included= employed by the City of Helsinki less than 4 years or incomplete data on occupational position.

For peer review only

Web-appendix 2. Comparison between those included* and those not included* in the present study, women

Women	Total		Included		Excluded	
	N	%	N	%	N	%
**Total**	16163	100.0	7308	100.0	8855	100.0
**Age**						
20 - 24	5609	34.7	2252	30.8	3357	37.9
25 - 29	6735	41.7	3152	43.1	3583	40.5
30 - 34	3819	23.6	1904	26.1	1915	21.6
**Language**						
Finnish	14028	86.8	6451	88.3	7577	85.6
Swedish	803	5.0	324	4.4	479	5.4
Other	1137	7.0	448	6.1	689	7.8
**Education**						
Basic education / Lower secondary	8785	54.4	3641	49.8	5144	58.1
Upper secondary	4420	27.3	2319	31.7	2101	23.7
Higher education	2958	18.3	1348	18.4	1610	18.2
**Occupational class**						
Managers or professionals	3674	22.7	1679	23.0	1995	22.5
Semi-professionals	2833	17.5	1538	21.0	1295	14.6
Routine non-manual workers	7561	46.8	3385	46.3	4176	47.2
Manual workers	1875	11.6	706	9.7	1169	13.2
**Working hours per week**						
32–45 h/wk	12678	78.4	5891	80.6	6787	76.6
<32 h/wk	3485	21.6	1417	19.4	2068	23.4
**Type of employment contract**						
Permanent contract	13059	80.8	6422	87.9	6637	75.0
Other contract type	3104	19.2	886	12.1	2218	25.0

*Included = employed by the City of Helsinki for at least 4 years and complete data on occupational position, Not included= employed by the City of Helsinki less than 4 years or incomplete data on occupational position.

For peer review only

Web-appendix 3. Multinomial logistic regression on occupational class with reversed order of categories as a determinant of occupational health service (OHS) trajectories among 9762 City of Helsinki employees aged 20-34 years. Results are based on register data covering the years from 2004 to 2017.

	OHS trajectory comparison	
	Low/increasing vs. No OHS visits	
	Model 1*	Model 2**
**Men**		
**Occupational class**		
Worker occupations***	1.00	1.00
Semi-professionals	1.02 (0.72-1.44)	1.04 (0.72-1.50)
Managers or professionals	0.65 (0.49-0.88)	0.79 (0.55-1.13)
**Women**		
**Occupational class**		
Worker occupations	1.00	1.00
Semi-professionals	0.83 (0.70-0.99)	0.84 (0.69-1.02)
Managers or professionals	0.67 (0.56-0.79)	0.70 (0.56-0.89)
	Low/decreasing vs. No OHS visits	
	Model 1*	Model 2**
**Men**		
**Occupational class**		
Worker occupations	1.00	1.00
Semi-professionals	1.03 (0.74-1.43)	0.96 (0.68-1.36)
Managers or professionals	0.78 (0.60-1.02)	0.66 (0.47-0.93)
**Women**		
**Occupational class**		
Worker occupations	1.00	1.00
Semi-professionals	1.12 (0.97-1.30)	1.02 (0.86-1.22)
Managers or professionals	0.76 (0.65-0.88)	0.60 (0.48-0.74)
	High/recurrent vs. No OHS visits	
	Model 1*	Model 2**
**Men**		
**Occupational class**		
Worker occupations	1.00	1.00
Semi-professionals	0.70 (0.43-1.15)	0.80 (0.47-1.34)
Managers or professionals	0.21 (0.13-0.38)	0.31 (0.16-0.58)
**Women**		
**Occupational class**		
Worker occupations	1.00	1.00
Semi-professionals	0.81 (0.66-0.99)	0.98 (0.77-1.24)
Managers or professionals	0.38 (0.30-0.49)	0.42 (0.31-0.59)

* Model adjusted for age and language ** Model 1 adjusted for education *** Combined class for routine non-manual employees and manual workers

For peer review only

STROBE Statement—checklist of items that should be included in reports of observational studies

Young Employees' Trajectories and Occupational Class Differences in Utilization of Primary Care Services Provided by Occupational Health Service

Hilla Sumanen, PhD
Jaakko Harkko, PhD
Kustaa Piha, PhD
Olli Pietiläinen, MSc
Ossi Rahkonen, Professor
Anne Kouvonen, Professor

	Item No	Recommendation	Done, page
**Title and abstract**	1	(*a*) Indicate the study's design with a commonly used term in the title or the abstract	3
		(*b*) Provide in the abstract an informative and balanced summary of what was done and what was found	3
**Introduction**			
Background/rationale	2	Explain the scientific background and rationale for the investigation being reported	4-5
Objectives	3	State specific objectives, including any prespecified hypotheses	5
**Methods**			
Study design	4	Present key elements of study design early in the paper	5
Setting	5	Describe the setting, locations, and relevant dates, including periods of recruitment, exposure, follow-up, and data collection	5-6
Participants	6	(*a*) *Cohort study*—Give the eligibility criteria, and the sources and methods of selection of participants. Describe methods of follow-up<br>*Case-control study*—Give the eligibility criteria, and the sources and methods of case ascertainment and control selection. Give the rationale for the choice of cases and controls<br>*Cross-sectional study*—Give the eligibility criteria, and the sources and methods of selection of participants	5-6
		(*b*) *Cohort study*—For matched studies, give matching criteria and number of exposed and unexposed<br>*Case-control study*—For matched studies, give matching criteria and the number of controls per case	
Variables	7	Clearly define all outcomes, exposures, predictors, potential confounders, and effect modifiers. Give diagnostic criteria, if applicable	6-7
Data sources/ measurement	8*	For each variable of interest, give sources of data and details of methods of assessment (measurement). Describe comparability of assessment methods if there is more than one group	6
Bias	9	Describe any efforts to address potential sources of bias	7, 14-

For peer review only - http://bmjopen.bmj.com/site/about/guidelines.xhtml

			15
Study size	10	Explain how the study size was arrived at	5-6
Quantitative variables	11	Explain how quantitative variables were handled in the analyses. If applicable, describe which groupings were chosen and why	7
Statistical methods	12	(*a*) Describe all statistical methods, including those used to control for confounding	7
		(*b*) Describe any methods used to examine subgroups and interactions	7
		(*c*) Explain how missing data were addressed	
		(*d*) *Cohort study*—If applicable, explain how loss to follow-up was addressed<br>*Case-control study*—If applicable, explain how matching of cases and controls was addressed<br>*Cross-sectional study*—If applicable, describe analytical methods taking account of sampling strategy	
		(*e*) Describe any sensitivity analyses	10

**Results**			**Done, page**
Participants	13*	(a) Report numbers of individuals at each stage of study—eg numbers potentially eligible, examined for eligibility, confirmed eligible, included in the study, completing follow-up, and analysed	5-8
		(b) Give reasons for non-participation at each stage	
		(c) Consider use of a flow diagram	
Descriptive data	14*	(a) Give characteristics of study participants (eg demographic, clinical, social) and information on exposures and potential confounders	7-8
		(b) Indicate number of participants with missing data for each variable of interest	
		(c) *Cohort study*—Summarise follow-up time (eg, average and total amount)	5-6, 8
Outcome data	15*	*Cohort study*—Report numbers of outcome events or summary measures over time	6, 8
		Case-control study—Report numbers in each exposure category, or summary measures of exposure	
		Cross-sectional study—Report numbers of outcome events or summary measures	
Main results	16	(*a*) Give unadjusted estimates and, if applicable, confounder-adjusted estimates and their precision (eg, 95% confidence interval). Make clear which confounders were adjusted for and why they were included	7-10
		(*b*) Report category boundaries when continuous variables were categorized	
		(*c*) If relevant, consider translating estimates of relative risk into absolute risk for a meaningful time period	
Other analyses	17	Report other analyses done—eg analyses of subgroups and interactions, and sensitivity analyses	10
**Discussion**			
Key results	18	Summarise key results with reference to study objectives	10-11
Limitations	19	Discuss limitations of the study, taking into account sources of potential bias or imprecision. Discuss both direction and magnitude of any potential bias	14-15
Interpretation	20	Give a cautious overall interpretation of results considering objectives, limitations, multiplicity of analyses, results from similar studies, and other relevant evidence	10-15
Generalisability	21	Discuss the generalisability (external validity) of the study results	10-15
**Other information**			

For peer review only - http://bmjopen.bmj.com/site/about/guidelines.xhtml

Funding	22	Give the source of funding and the role of the funders for the present study and, if applicable, for the original study on which the present article is based	16

*Give information separately for cases and controls in case-control studies and, if applicable, for exposed and unexposed groups in cohort and cross-sectional studies.

Note: An Explanation and Elaboration article discusses each checklist item and gives methodological background and published examples of transparent reporting. The STROBE checklist is best used in conjunction with this article (freely available on the Web sites of PLoS Medicine at http://www.plosmedicine.org/, Annals of Internal Medicine at http://www.annals.org/, and Epidemiology at http://www.epidem.com/). Information on the STROBE Initiative is available at www.strobe-statement.org.

For peer review only

For peer review only - http://bmjopen.bmj.com/site/about/guidelines.xhtml
